# Synthetic Microbial Cocultivation for Targeted Production of Odd-Chain Carboxylates and Alcohols from Carbon Monoxide

**DOI:** 10.1021/acs.est.4c14794

**Published:** 2025-08-27

**Authors:** Ivette Parera Olm, Sara Benito-Vaquerizo, Charles Dubaere, Vitor A. P. Martins dos Santos, Maria Suarez-Diez, Diana Z. Sousa

**Affiliations:** † Laboratory of Microbiology, 4508 Wageningen University & Research, 6708 WE Wageningen, The Netherlands; ‡ Centre for Living Technologies, Eindhoven-Wageningen-Utrecht Alliance, 3584 CB Utrecht, The Netherlands; § Laboratory of Systems and Synthetic Biology, Wageningen University & Research, 6708 WE Wageningen, The Netherlands

**Keywords:** syngas fermentation, microbial communities, acetogens, chain elongation, genome-scale metabolic model, community modeling

## Abstract

Microbial fermentation of syngas (CO, H_2_, CO_2_) using acetogens is a promising route for the revalorisation of one-carbon feedstocks. However, product diversification from syngas using pure cultures of these microorganisms remains a challenge. Here, we present a synthetic microbial community comprising the acetogen *Acetobacterium wieringae* JM, the propionigenic bacterium *Anaerotignum neopropionicum* and the chain elongator *Clostridium kluyveri*, which collectively produce odd- and even-chain carboxylic acids and higher alcohols from CO/CO_2_. In batch bioreactors, metabolite cross-feeding within the community enabled the production of valerate (0.61 g L^–1^) and pentanol (0.33 g L^–1^), which are rare products in CO-fermenting systems. Chemostat experiments showed a metabolic shift induced in the acetogen by the ethanol-consuming species. Furthermore, construction of the genome-scale metabolic model (GEM) of *A. wieringae* JM and a community model of the triculture allowed us to predict the performance of the culture in continuous (steady-state) process. Simulations using flux balance analysis predicted a feasible triculture with *A. wieringae* JM dominating the community, and provided insights into the effect of H_2_ supplementation on the product spectrum. The results of our study underscore the potential of synthetic microbial communities for syngas fermentation, with genome-scale metabolic modeling serving as a powerful tool to identify metabolic shifts and guide experimental design.

## Graphical abstract

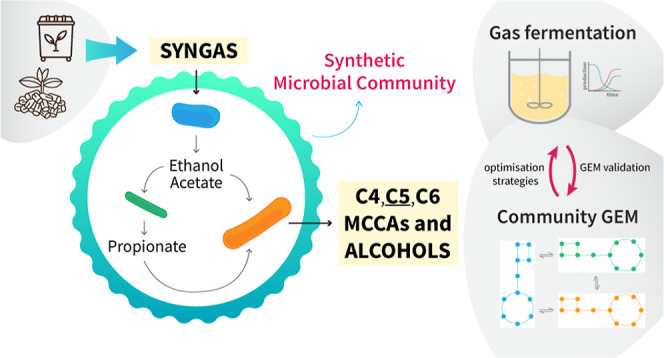

## Introduction

The pressing need to reduce fossil fuel dependency and mitigate greenhouse gas emissions has driven interest in the production of biochemicals from one-carbon (C1) feedstocks.^1^ Syngas is a highly versatile C1 feedstock consisting mainly of CO, H_2_ and CO_2_ that is typically derived from the gasification of lignocellulosic or other organic residues, and can be converted into valuable biochemicals via microbial fermentation.^2^ Industrial CO-rich waste gases, such as from steel mills, can also be repurposed for fermentation,^3,4^ and solar-powered coelectrolysis of CO_2_ and steam offers a route to “white” syngas.^5–7^ Syngas fermentation relies on acetogenic bacteria, including *Clostridium* and *Acetobacterium* species, which metabolize CO_2_ using electron donors such as CO or H_2_ via the reductive acetyl-CoA (Wood–Ljungdahl) pathway, primarily producing acetate and ethanol.^8^ Acetogens have been extensively studied for this process,^9,10^ with significant genetic engineering efforts done in the past decade to expand their range of products.^11,12^ However, genetic manipulation of acetogens remains challenging due to native restriction–modification barriers and inadequate efficiency of transformation protocols.^13–15^ Moreover, their energy-constrained metabolism restricts the synthesis of longer-chain hydrocarbons and other ATP-demanding products.^16^

Microbial communities, whether open mixed cultures or synthetic consortia, offer another avenue to diversify the product range of syngas fermentation.^17^ Synthetic cocultures, in particular, can be rationally designed to steer fermentation toward the selective production of target compounds.^18,19^ A well-known example is the cocultivation of acetogens with *Clostridium kluyveri* to produce medium-chain carboxylic acids (MCCAs), such as caproate, via chain elongation.^20^ In this process, *C. kluyveri* uses syngas-derived ethanol and acetate as substrates, extending the carboxylate carbon chain by two carbons per cycle (i.e., acetate to butyrate, and butyrate to caproate) through the reverse β-oxidation pathway.^21,22^ Higher alcohols (≥C3) can also be produced in these cocultures during a solventogenic phase, where acetogens couple CO oxidation to the reduction of carboxylic acids.^23^ Butanol and hexanol at concentrations of up to 1 g L^–1^ have been reported for cocultures with acetogens such as *Clostridium autoethanogenum*,^24^ *Clostridium aceticum* ^25^ or *Clostridium carboxidivorans*,^26,27^ with even higher titers achievable in systems with product extraction.^28^ Since acetate and ethanol are the main intermediates in this process, most studies integrating syngas fermentation and chain elongation have focused on even-carbon end products.^20,23^ Although *C. kluyveri* can also metabolize odd-chain substrates such as propionate or propanol,^29^ these intermediates are typically absent or present in minimal amounts in these systems. As a result, odd-chain MCCAs (i.e., valerate, heptanoate) are usually detected only in trace quantities.^30,31^ Notably, He et al. reported concentrations of these products of up to 0.2 g L^–1^ using a mixed culture in a system with gas recirculation.^32^ However, the long acclimation period (>100 days) to CO and difficulties in suppressing methanogenesis underscore major limitations of syngas fermentation with mixed cultures.

Previously, we established a synthetic coculture comprising *C. kluyveri* and the propionigenic bacterium *Anaerotignum neopropionicum* that produced valerate and heptanoate from ethanol and CO_2_.^33^ *A. neopropionicum* metabolizes these substrates via the acrylate pathway,^34,35^ generating propionate and acetate that are subsequently used by *C. kluyveri* for ethanol-driven chain elongation. In the present work, we aimed to integrate odd-chain elongation and syngas fermentation into a single bioprocess by incorporating the acetogen *Acetobacterium wieringae* JM. Unlike the previously cited acetogens, which are acidophilic,^9^ this strain grows efficiently at circumneutral pH,^36^ similarly to *A. neopropionicum* ^35^ and *C. kluyveri*.^37^ We hypothesized that, in a triculture, propionate formed by *A. neopropionicum* from CO-derived ethanol would enable the generation of odd-chain end products (Figure 1). As a proof of concept, we evaluated the productivity of the synthetic triculture (*A. wieringae* JM –*A. neopropionicum*–*C. kluyveri*) in batch bioreactor fermentation under continuous CO feeding. Additionally, continuous chemostat cultivations were conducted to assess how cocultivation influences the product spectrum and gas consumption by the acetogen when paired with each strain.

**Figure 1:**
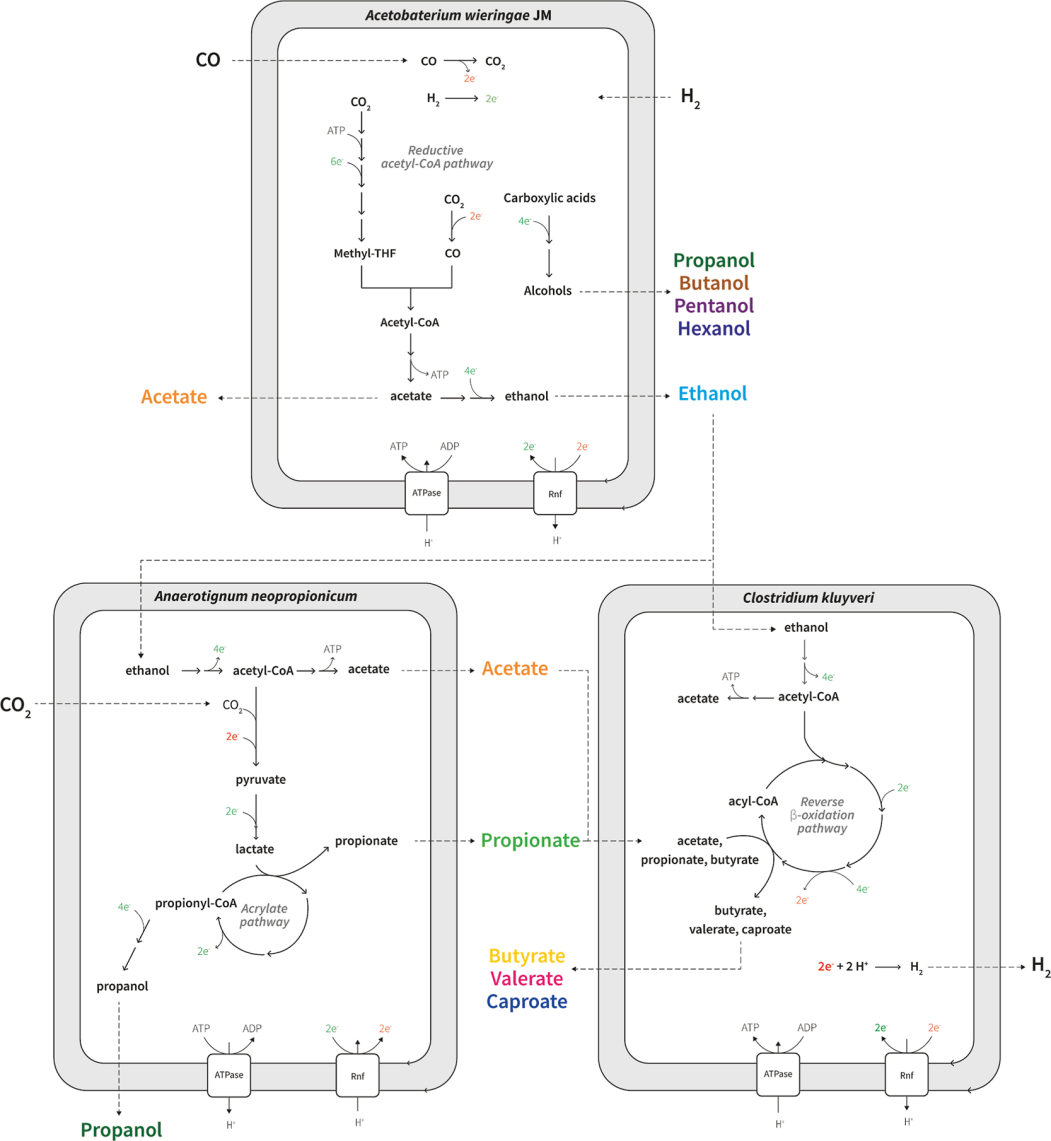
Schematic representation of the syngas-fermenting synthetic triculture comprising *A. wieringae* JM, *A. neopropionicum* and *C. kluyveri*. Simplified metabolic pathways are depicted, highlighting key conversions and metabolites. Electrons in red represent ferredoxin. ATPase: ATPase/synthase; Rnf: H^+^/Na^+^-translocating ferredoxin:NAD^+^ oxidoreductase.

In addition to the experimental work, we employed genome-scale metabolic modeling and community flux balance analysis (cFBA) to provide a predictive framework for establishing the triculture in continuous cultivation. Genome-scale metabolic models (GEMs) are mathematical representations of the genome-encoded metabolic potential of individual organisms and can be integrated to construct community models that capture interspecies interactions.^38–42^ cFBA is an extension of the flux balance analysis (FBA) modeling approach that assumes steady-state conditions and incorporates the relative abundances of strains in cocultures.^42^ This makes it well-suited for assessing the impact of continuous cultivation parameters on community metabolism and process productivity. The combination of GEMs and cFBA has been used to simulate microbial community growth and predict metabolic scenarios, such as the production of target compounds, thereby informing bioprocess design.^38,39,43^ Here, we built the first GEM of *A. wieringae* JM (AWIEJM-GEM) and combined it with the existing models of *C. kluyveri* ^44^ and *A. neopropionicum* ^34^ to generate a community GEM of the triculture. Using cFBA, we identified species ratios and bioreactor dilution rates that supported a feasible triculture model, and evaluated the effect of species ratios and H_2_ supplementation on the end-product profile. Together, the experimental and computational efforts in this study demonstrate the targeted production of odd-chain MCCAs and higher alcohols from syngas using a synthetic coculture, and suggest experimental conditions for process optimization.

## Materials and Methods

### Microbial Strains and Cultivation

*C. kluyveri* DSM 555^T^ and *A. neopropionicum* DSM 3847^T^ were purchased from DSMZ (German Collection of Microorganisms and Cell Cultures; Braunschweig, Germany). *A. wieringae* strain JM is an isolate from our own culture collection.^36^ *C. kluyveri* and *A. neopropionicum* were cultivated in bicarbonate-buffered medium (40 mM, pH 7) containing (per liter): 0.9 g NH_4_Cl, 0.3 g NaCl, 0.8 g KCl, 0.2 g KH_2_PO_4_, 0.4 g K_2_HPO_4_, 0.2 MgSO_4_ × 7 H_2_O, 0.04 CaCl_2_ × 2 H_2_O, 3.36 g NaHCO_3_, 10 mL trace element solution from DSMZ medium 318 (Table S1), 1 mL vitamin solution (Table S2), 0.5 g yeast extract, 0.24 g Na_2_Sx × H_2_O (*x* = 9–11) and 0.5 g l-cysteine–HCl as reducing agents, and 0.5 mg resazurin as redox indicator. Cultures were grown in 117 mL serum bottles containing 50 mL medium, and the headspace of bottles was pressurized with N_2_/CO_2_ (80:20% v/v, 150 kPa). Cultures of *A. neopropionicum* were supplemented with ethanol (50 mM) and incubated statically at 30 °C, while *C. kluyveri* was grown on ethanol (90 mM) and acetate (75 mM) and incubated at 37 °C. *A. wieringae* JM was cultivated in phosphate-buffered medium (30 mM, pH 7) containing (per liter): 1 g NH_4_Cl, 0.7 g NaCl, 2.8 g KH_2_PO_4_, 1.3 g Na_2_HPO_4_, 0.2 g MgSO_4_·7H_2_O, 0.04 g CaCl_2_·2H_2_O, 10 mL modified trace element solution from ATCC medium 1754 (Table S3), 1 mL vitamin solution (Table S2), 0.5 g yeast extract, 0.75 g l-cysteine–HCl as reducing agent and 0.5 mg resazurin as redox indicator. *A. wieringae* JM was grown with 100% CO (v/v) in the headspace (170 kPa) as substrate; cultures were incubated at 30 °C, shaking (150 rpm) after 24 h statically.

### Batch Bioreactor Cultivation with Continuous Gas Supply

The triculture comprising *A. wieringae* JM, *A. neopropionicum* and *C. kluyveri* was established in batch bioreactors with continuous gas supply. Fermentations were carried out in four replicate 1.3-L Applikon Bio bioreactors (Getinge, Sweden) with a working volume of 700 mL. The bioreactors were fitted with temperature, pH and redox sensors. Gas inflow (CO, N_2_, CO_2_) was independently regulated using mass flow controllers (Getinge, Sweden). The medium contained (per liter): 1 g NH_4_Cl, 0.8 g NaCl, 0.1 g KH_2_PO_4_, 0.2 g MgSO_4_ × 7 H_2_O, 0.02 g CaCl_2_ × 2 H_2_O, 10 mL modified trace element solution from ATCC medium 1754 (Table S3), 1 mL vitamin solution (Table S2), 75 mM 2-(*N*-Morpholino)­ethanesulfonic acid (MES), 0.5 g yeast extract, 0.75 g l-cysteine–HCl and 0.5 mg resazurin. To prevent excessive foaming, 0.25 mL of 1% Antifoam 204 (A6424; Sigma-Aldrich) was aseptically added to all bioreactors at 86 h of operation, when foam accumulation became evident. The temperature was maintained at 30 °C, and the pH was controlled at 7 via the automated addition of 3 M NaOH and 2 M HCl. The startup procedure was done as follows: the autoclaved reactor vessels containing medium salts, trace elements, MES and resazurin were sparged with N_2_ (1.5 L h^–1^) for ≈2 h to establish anaerobic conditions. Then, yeast extract, vitamins and l-cysteine–HCl were added to the medium from anaerobic, sterile stock solutions, and the gas inflow was set to N_2_/CO_2_ (75:25% v/v, 16.8 mL min^–1^). When the redox potential had stabilized at ≈−300 mV, *C. kluyveri* and *A. neopropionicum* were inoculated from active precultures (5% v/v and 7% v/v, respectively). Ethanol (20 mM) was added to support initial growth of these strains and to prevent the acetogen from overtaking the culture, as observed in preliminary experiments (not shown). After 41.8 h, when cell growth was observed (indicated by an increase in optical density at 600 nm), *A. wieringae* JM was inoculated (1% v/v) and the CO feeding was set to 11.2 mL min^–1^. This resulted in a total gas composition of 40% CO, 45% N_2_ and 15% CO_2_ (v/v) at a flow rate of 28 mL min^–1^ (0.04 vvm), which was maintained for the remainder of the fermentation. Agitation was provided by two six-bladed impellers; the stirring was initially set at 100 rpm and later increased to 150, 250, 300, and 400 rpm (at 86, 95, 117.8, and 209 h, respectively). Low to moderate stirring was applied to prevent CO saturation in the liquid; while *A. neopropionicum* is not affected by CO during growth on ethanol (data not shown), inhibition has been reported for *C. kluyveri*.^24^

Liquid samples were routinely taken for analysis of cell density, soluble products and microscopy inspection. Culture samples were taken at the end (183 h) for cell dry weight (CDW) determination. Gas outflow rates were regularly measured using an ADM flow meter (Agilent, United States). Automated online sampling of the headspace was done via a gas chromatograph (GC; Compact GC 4.0, Global Analyzer Solutions, The Netherlands) coupled to the bioreactor system, equipped with two channels and a thermal conductivity detector (TCD). CO and H_2_ were analyzed in a Molsieve 5A column operated at 50 °C, preceded by an Rt-Q-BOND precolumn. CO_2_ was quantified in an Rt-Q-BOND column operating at 50 °C. Argon was used as carrier gas.

### Chemostat Bioreactor Experiment with Continuous Gas Supply

A chemostat experiment was carried out to examine the physiological response of *A. wieringae* JM to the presence of either ethanol-consuming species. The experiment was conducted in two parallel 0.5-L BioXplorer 400P stirred-tank bioreactors (H.E.L. Group, Borehamwood, United Kingdom) with 400 mL working volume. The systems were equipped with temperature, pH and redox sensors. Reactor 1 (R1) was used to study the coculture of *A. wieringae* JM with *C. kluyveri*, while Reactor 2 (R2) was employed for the coculture of *A. wieringae* JM with *A. neopropionicum*. The medium used was as described above for *A. wieringae* JM, amended with 0.005% (v/v) Antifoam 204 to prevent excessive foaming. The startup procedure followed the steps described above for the batch bioreactor experiment, with the following differences. The inflow gas composition and flow rate were set to CO/N_2_ (70:30% v/v) and 4 mL min^–1^ (0.01 vvm), respectively, regulated by individual mass flow controllers. When the redox potential had stabilized below −300 mV, cultivation was initiated in the two bioreactors by inoculating *A. wieringae* JM (5%, v/v) from an active preculture. The temperature was controlled at 30 °C, and the pH maintained at 7 via the automated addition of 3 M KOH. A brief batch phase lasting 3.8 days was established prior to continuous operation to allow for growth of *A. wieringae* JM. Continuous operation was started by aseptically supplying fresh medium from a 20-L tank via a peristaltic pump, adjusted to a hydraulic retention time (HRT) of 48 h (dilution rate, *D* = 0.021 h^–1^). Through the operation, the medium tank was flushed with N_2_ to maintain anaerobic conditions.

In both bioreactors, the experimental procedure during continuous operation involved two phases: first, a monoculture of *A. wieringae* JM (therefore, R1 and R2 are regarded as duplicates during this stage), and second, a coculture with either *C. kluyveri* (in R1) or *A. neopropionicum* (in R2). The partner strains were inoculated on day 60 with 5% (v/v) of actively growing precultures. Each phase (mono- and coculture) was deemed complete once steady state conditions were achieved, characterized by stable biomass and acetate concentrations (±5–7%) over a period exceeding three HRTs. Deviations of up to 14% occurred occasionally due to technical issues, which are noted in the text. Agitation (via magnetically driven captive impeller) was set at 350 rpm for the initial batch phase, followed by increases to 500 rpm (on day 5.8) and 700 rpm (on day 23.7) during continuous operation. Temporary fluctuations of the stirring speed occurred at various points due to system failures, as indicated in the text. Gas outflow rates were regularly measured using an ADM flow meter (Agilent, United States). Liquid outflow rates were regularly measured to determine the actual HRT. Liquid and headspace samples were routinely collected for further analysis.

### Batch Bottle Tests with *A. wieringae* JM

Batch experiments in serum bottles were conducted to test the ability of *A. wieringae* JM to reduce C3–C6 carboxylic acids to alcohols with CO. Cultivation was done in the medium and conditions described above for this strain (30 °C, pH 7), with 100% CO in the headspace (170 kPa). Bottles were supplemented with 20 mM of propionate, butyrate, valerate or caproate in their sodium salt form. Control bottles were not supplemented. Liquid and headspace samples were taken at the start and the end of cultivation for analysis of CDW, soluble compounds and gas composition. Control cultures and those with propionate and butyrate were stopped after 9 days; cultures with valerate and caproate were ended after 28 days. All conditions were tested in triplicates.

### Analytical Techniques and Calculations

Microbial growth was determined based on optical density measurements at 600 nm (OD_600_) using a spectrophotometer (UV-1800, Shimadzu, Japan). CDW was determined by gravimetric analysis: pellets from a known culture volume (≈50 mL) were washed twice in deionized water, transferred into preweighed aluminum trays, dried overnight at 105 °C, and weighed again the next day.

Gas samples from the bottle and chemostat experiments were analyzed in a two-channel GC (Compact GC 4.0, Global Analyzer Solutions, The Netherlands) equipped with a thermal conductivity detector (TCD). CO and H_2_ were detected using a Molsieve 5A column operated at 100 °C and coupled to a Carboxen 1010 precolumn, while CO_2_ was analyzed in a Rt-Q-BOND column operated at 60 °C. In both channels, argon was used as carrier gas. Concentrations of ethanol, propanol and C2–C4 carboxylic acids in liquid samples (from all experiments) were determined by high-performance liquid chromatography (HPLC; LC-2030C, Shimadzu, Japan). The apparatus was equipped with a Shodex SH1821 column operating at 60 °C, and 0.01 N H_2_SO_4_ was used as eluent (1 mL min^–1^). Amounts detected in a concentration <0.3 mM could not be accurately quantified and are considered traces. Valerate, caproate and C4–C6 alcohols were measured with a GC2010-Pro (Shimadzu, Japan) equipped with a HS-20 headspace autosampler. The GC carried a DB-WAX Ultra Inert column (Agilent, USA) that operated with a temperature gradient of 60 °C–220 °C at a rate of 25 °C min^–1^. Carrier gas was nitrogen. Samples were prepared in 10 mL headspace vials with 100 μL of sample, 100 μL internal standard solution (10 mM 2-ethylbutyric acid) and 200 μL acidified desalting solution (0.4 N H_2_SO_4_ in 20% NaCl). Vials were transferred to the oven of the autosampler that was held at 60 °C. After an equilibrating time of 5 min, a sample of the headspace was brought onto the column. Detector was a FID detector. The Chromeleon data analysis software (Thermo Fisher Scientific), version 7.2.9, was used for both GC and HPLC peak analysis.

Consumption and production rates from chemostat bioreactor cultivations were calculated from mass balances, using concentrations, partial pressures, volumes and/or flow rates measured or set as described above. The percentage of CO consumption was calculated dividing the calculated CO consumption rate by the CO inflow rate (set and measured value).

### Reconstruction and Validation of AWIEJM-GEM

The genome-scale metabolic model (GEM) of *A. wieringae* JM (AWIEJM-GEM) was reconstructed employing an orthology-based approach and drawing from the standard-GEM git-based template recently proposed by the community for standardization of models and best practices.^45^ The GEM of *C. autoethanogenum* (iCLAU786)^46^ was used as scaffold, with further refinement based on the model of *Acetobacterium woodii* ^47^ and the genomic content of *Acetobacterium wieringae* ^T^ and other *Acetobacterium* species.^36,48^ Detailed methodology is available in the Supporting Information. The model is available in several formats together with a MEMOTE^49^ quality report in the GitLab repository https://gitlab.com/wurssb/Modelling/AWIEJM-GEM, and in a COMBINE archive in BioModels^50,51^ with a FROG report,^52^ under the identifier MODEL2310100001. The model underwent both qualitative and quantitative validation, as detailed in the Supporting Information. In short, qualitative validation was done employing FBA to evaluate growth phenotypes on 11 carbon sources, the majority of which have been experimentally tested. For quantitative validation, model-predicted production rates of acetate and ethanol during growth on CO were compared with those measured in duplicate monoculture phases of chemostat fermentations of *A. wieringae* JM performed in this study.

### Construction of the Community GEM of the Tri-culture

The community GEM of the triculture was constructed by combining the GEMs of *A. wieringae* JM (AWIEJM-GEM; this study), *A. neopropionicum* (iANEO_607)^34^ and *C. kluyveri* (iCKL708).^44^ The methodology followed was as described previously^38,39^ and is depicted in the Supporting Information (Figure S3). Briefly, each species was designated to an intracellular compartment, namely “Cytosol_AW”, “Cytosol_AN”, and ‘Cytosol_CK”—with “aw”, “an” and “ck” as the respective identifiers (id). The extracellular compartment (“_e”) was shared by the three species. Intracellular metabolites were labeled with the id of the metabolite and the corresponding compartment id (e.g., acetate_aw). Reactions and metabolites that were defined as extracellular (“_e”) in the single-species models were also defined as extracellular in the triculture model. Extracellular reactions and metabolites originally present in more than one species were given the same nomenclature in the triculture model. Similarly, repeated extracellular reactions across the individual models were unified to be unique in the triculture model. The exchange of metabolites in the model is described by transport reactions from the intracellular compartment of the species that produces such metabolite to the extracellular compartment, and from the extracellular compartment to the intracellular compartment of the species that metabolizes it (Figure S3; reaction type 2). The biomass synthesis reaction of each of the species are denoted by the id: “Biomass_aw”, “Biomass_an” and “Biomass_ck” (Figure S3; reaction type 5). An additional reaction was created to represent the community biomass (“EX_Biomass_e”), which combined the individual biomass reactions with the stoichiometry corresponding to the biomass species ratio. The triculture model was deposited as SBML level 3 version 1 in our Git repository (https://gitlab.com/wurssb/Modelling/triculture_aw_an_ck).

### Simulations of the Community GEM Using cFBA

FBA was used to predict the triculture performance when established in continuous mode (e.g., in chemostat). For that, we evaluated the feasibility of the triculture in a range of combinations of species ratios (0.1–0.8) and growth rates (0.005–0.08 h^–1^) during growth on CO. As described in the modeling framework (Supporting Information), specific fluxes (mmol g_CDW_^–1^ h^–1^) were replaced by environmental fluxes (mmol h^–1^), considering the biomass density of each species (g L^–1^) and the culture volume (L). The total biomass of the community was estimated based on biomass measurements from monoculture chemostat cultivation of *A. wieringae* JM (0.32 g). For that total biomass, we assessed growth with all the combinations of growth rates and species ratios. The community biomass reaction is defined as the sum of the biomass contribution of each species, that is the sum of the biomass density of each species obtained from the indicated species ratio and community biomass, multiplied by the growth rate. Thus, for every species ratio and growth rate assessed, the stoichiometry of the community biomass reaction was changed according to the species ratios, and the value of community biomass reaction was constrained to the community biomass multiplied by the specified growth rate (Supporting Information). The biomass of each species was also constrained to the corresponding species ratio multiplied by the community biomass and the same growth rate of the community. The lower bound of the CO uptake rate was constrained between −3.5 mmol h^–1^ and −1 mmol h^–1^, based on CO inflow rates measured in chemostat cultivation of *A. wieringae* JM (this study). The community biomass was defined as the objective function. The triculture model was considered feasible when FBA led to a solution under each specified condition. We also explored the metabolite profile of CO fermentation at a fix growth rate (0.02 h^–1^) and with biomass species ratios where *A. wieringae* JM was the most abundant species in the community. The solution space and the set of fluxes compatible with the measured constraints were sampled using the sample function in the flux_analysis submodule of COBRApy.

To evaluate the impact of H_2_ as additional energy source on the production of carboxylic acids by the triculture, H_2_ uptake rates were varied from −5 to −2 mmol h^1^, with the growth rate fixed at 0.02 h^–1^. Two species ratios were evaluated: 5:3:2 and 7:1:2 (*A. wieringae* JM/*A. neopropionicum*/*C. kluyveri*), where the acetogen was the most abundant, resembling bioreactor experiments. The total biomass was fixed to 0.32 g, as in chemostat experiments with the acetogen. The CO uptake rate was constrained between −3.5 mmol h^–1^ and −2.5 mmol h^–1^. 5000 iterations were generated for each condition, using flux sampling. Simulations can be found in the Git repository.

## Results and Discussion

### CO-Derived Propionate Supports the Production of Valerate and Pentanol in the Synthetic Triculture

The synthetic triculture was established in quadruplicate batch bioreactors continuously fed with CO/N_2_/CO_2_ (40:45:15% v/v, 101 kPa), maintained at pH 7 and with stepwise increases in agitation. Upon inoculation of *A. wieringae* JM and start of the CO feed (first dashed line; Figure 2), cell density increased rapidly, reaching a maximum OD_600_ of 3.3 ± 0.5 at 135 h. Concomitantly, the production of C2–C6 carboxylic acids was observed, suggesting active growth and community establishment during the early phase of cultivation. Increased agitation from 100 to 150 and 250 rpm had minimal impact on the product profiles except for acetate, indicating predominant growth of the acetogen. This was supported by microscopy, which confirmed the presence of the three strains in the culture, with *A. wieringae* JM being dominant (Figure S1). Metabolic activity continued until 209 h despite stagnant cell density, suggesting a shift to maintenance metabolism in the later stage of fermentation. Final biomass concentration was 1.06 ± 0.05 g L^–1^. CO consumption remained below 25%, as indicated by CO partial pressures of 30–40 kPa (Figure S2b). A small H_2_ peak (0.15 kPa) was observed at the start (Figure S2a), likely due to initial growth of *C. kluyveri*, but H_2_ partial pressures remained very low for the rest of the cultivation despite evident metabolic activity of *C. kluyveri*, suggesting H_2_ consumption by *A. wieringae* JM.^36^

**Figure 2:**
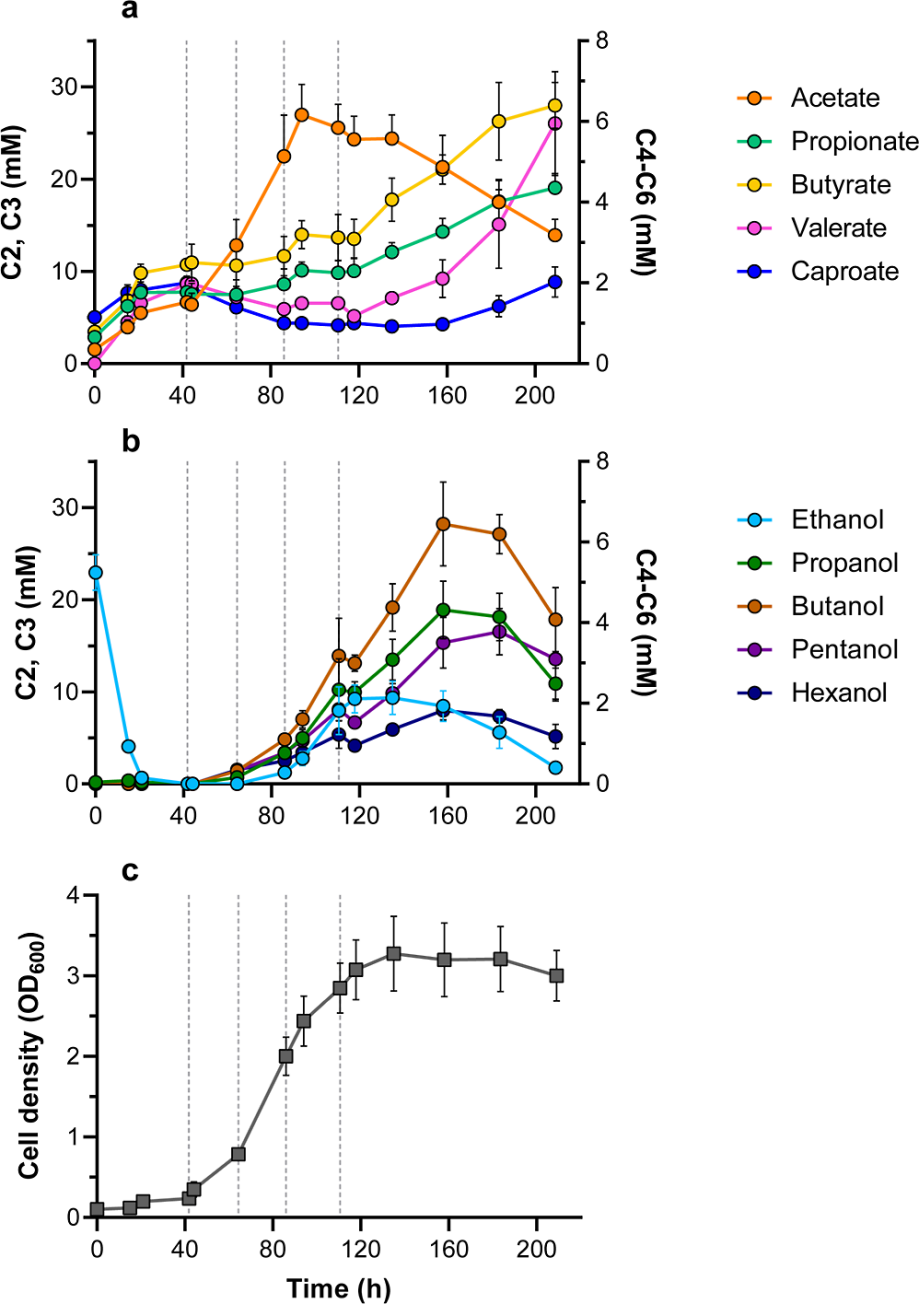
Cultivation of the synthetic triculture in batch bioreactor with continuous CO supply. (a,b) Concentration of carboxylic acids and alcohols, respectively. (c) Cell density. The first dashed line corresponds to the inoculation of *A. wieringae* JM and start of the CO inflow, with agitation at 100 rpm. The subsequent dashed lines indicate increases of the agitation to 150, 250, and 300 rpm. Data shown represent averages from quadruplicate bioreactors, with error bars indicating standard deviations.

Accordingly with acetogen dominance, acetate was the main product (max. 27.0 ± 3.3 mM), exceeding MCCAs concentrations by an order of magnitude (2–7 mM; Figure 2a). Propionate was the second most abundant carboxylic acid (19.1 ± 2.6 mM at 209 h), indicating significant activity of *A. neopropionicum*, supported by its moderate abundance in the culture (Figure S1). We estimate that ≈9 mM propionate and ≈8 mM acetate were produced by this strain via consumption of the 20 mM ethanol supplied (Figure 2b) and CO_2_. This aligns with a carbon balance (accounting for biomass and initial amounts of MCCAs produced by *C. kluyveri*) and observed propionate/acetate ratio in pure cultures.^34^ Subsequent propionate must have been derived from ethanol produced by *A. wieringae* JM from CO. Ethanol remained undetected during the initial phase, suggesting its rapid utilization by the ethanol-consuming species.

During the stationary phase (94–209 h), chain elongation intensified along with a gradual accumulation of higher alcohols. Agitation was raised to 300 rpm at the onset of this phase (fourth dashed line in Figure 2), promoting acetate reduction to ethanol by *A. wieringae* JM and overall product formation. Interestingly, chain elongation and solventogenesis took place simultaneously; likely, the consumption of carboxylic acids as they were reduced to alcohols (see discussion below) favored propionigenesis and chain elongation, both favored at the neutral pH of the fermentation.^22,35^ Butyrate (6.4 ± 0.6 mM) and valerate (6.0 ± 1.3 mM) were the most abundant MCCAs at the end of the fermentation, followed by caproate (2.0 ± 0.4 mM) and traces of heptanoate (<0.2 mM; not shown). Odd- and even-chain elongation occurred simultaneously, consistent with the behavior of *C. kluyveri* in pure cultures.^33,53^ Odd-chain elongation could have proceeded from ethanol and propionate, but also from propanol and acetate, as all these compounds were present in the system. However, butyrate production exceeded that of valerate, resulting in higher concentrations of C4 + C6 than C5 + C7 carboxylates (Figure 2a). This may reflect a higher enzymatic affinity of *C. kluyveri*’s reverse β-oxidation machinery for C2 over C3 intermediates. Moreover, previous studies have shown that, in the presence of propionate, *C. kluyveri* oxidizes excessive ethanol to acetate, increasing the acetate pool and the proportion of even-chain products.^33,53^ Despite this, the final valerate concentration we observed (6 mM; 0.61 g L^–1^) is, to our knowledge, the highest reported from CO/CO_2_ fermentation—a 3.6-fold increase over the maximum achieved with open mixed culture fermentation (0.17 g L^–1^).^32^

Higher alcohols were likely formed by *A. wieringae* JM via CO-driven reduction of carboxylic acids, a known trait of acetogens,^24,54,55^ and demonstrated for this strain here (see next section). A total of 30.7 mM higher alcohols (C3–C6) were produced, comprising 37.1% (mol/mol) of total products at 158 h (Figure 2b). Propanol, which might have also been formed by *C. kluyveri* or *A. neopropionicum*,^33,53^ was the most abundant alcohol (18.9 ± 3.1 mM) and it was produced alongside its precursor propionate. This suggests a steady propionate production in the system and, therefore, sustained metabolic activity of *A. neopropionicum*, as inferred before. Butanol (6.5 ± 1.0 mM), pentanol (3.8 ± 0.6 mM; peaking at 183 h) and hexanol (1.8 ± 0.2 mM) accounted for the remaining higher alcohols. Ethanol peaked at ≈10 mM, but it was progressively consumed. Actual alcohol production may have been slightly higher, as small amounts were detected in the outflow water bottle at the end, suggesting gas stripping losses. Pentanol, rarely reported in syngas fermentation,^30,31^ reached 3.8 mM (0.33 g L^–1^; Figure 2b), the highest reported concentration to date from syngas/CO fermentation. This highlights the triculture capability to sustain the endogenous production of odd-chain intermediates from CO.

Alcohol production typically follows the accumulation of carboxylic acids as a mechanism to mitigate acidification stress.^24,56^ The undissociated form of carboxylic acids is toxic to cells, since it crosses the membrane and can acidify the intracellular environment, ultimately inhibiting growth. Here, a total of 42.8 mM carboxylic acids accumulated at 94 h, when alcohol production began. However, the undissociated acid fraction was ≈0.27 mM (0.6%; at pH 7 and assuming an average p*K*_a_ of 4.83), below inhibitory thresholds observed in other Clostridia (0.5–1.5 mM).^24,57–59^ Thus, while this concentration could still be inhibitory to our strains, alcohol production may have also been triggered by nutrient depletion (as growth ceased), or enhanced CO transfer due to increased agitation. Notably, the observed solventogenesis by *A. wieringae* JM at neutral pH challenges the notion that acidic pH is essential for alcohol formation.^28,60,61^ Our findings indicate that conditions impeding growth (e.g., suboptimal pH, product toxicity, nutrient scarcity) and high availability of reducing equivalents (e.g., due to increased mass transfer rates) are determinant triggers for alcohol production, rather than acidic pH per se.^62^ Reports of solventogenesis at neutral pH by the acetogen *Alkalibaculum bacchi*,^54^ and by mixed cultures at pH 6.1–6.3 also support this view.^63^

### CO-Driven Reduction of C3–C6 Carboxylic Acids by *A. wieringae* JM

The ability of *A. wieringae* JM to produce higher alcohols from CO as electron donor was verified in batch tests supplemented with C3–C6 carboxylic acids (20 mM). CO consumption, products formed and cell density at the end of cultivations are summarized in Table 1. The analysis was validated by closed carbon and electron balances (91.7–101.4% and 85.4–114.1%, respectively).

**Table 1:** Reduction of C3–C6 Carboxylic Acids to Alcohols by *A. wieringae* JM^a^

carboxylate added (20 mM)	cell density (g L^–1^)	CO consumed (mmol) [%]	carboxylate consumed (mM) [%]	higher alcohol (mM)	acetate (mM)	ethanol (mM)	final pH
none (control)	0.52 ± 0.06	13.9 ± 0.3 [100 ± 0]			20.8 ± 2.2	17.6 ± 5.4	4.8
propionate	0.49 ± 0.10	14.1 ± 0.1 [100 ± 1]	9.1 ± 1.4 [48 ± 12]	8.3 ± 2.4 propanol	31.1 ± 8.9	11.0 ± 2.7	5.1
butyrate	0.46 ± 0.06	13.7 ± 0.5 [100 ± 2]	4.9 ± 2.2 [31 ± 13]	4.4 ± 2.2 butanol	28.5 ± 2.0	8.1 ± 5.3	5.0
valerate	0.14 ± 0.00	4.7 ± 0.6 [34 ± 4]	4.8 ± 0.4 [25 ± 0.4]	4.0 ± 0.7 pentanol	10.8 ± 0.1	1.8 ± 0.6	5.9
caproate	0.09 ± 0.01	3.2 ± 0.3 [23 ± 2]	1.7 ± 0.7 [10 ± 4]	2.1 ± 0.5 hexanol	9.8 ± 0.6	0.5 ± 0.1	6.2

a Data from batch incubations after 9 days (control, propionate and butyrate) or 28 days (valerate and caproate). Values shown are the average and standard deviations of biological triplicates.

*A. wieringae* JM reduced all C3–C6 carboxylic acids to their respective alcohols using CO as electron donor, albeit to varying degrees. CO depletion occurred only in control cultures and those with propionate or butyrate. Lower ethanol levels in the two latter cultures compared to the control (Table 1) suggest that direct availability of propionate and butyrate promoted early higher alcohol formation, limiting acidification and the subsequent need for ethanol as a counteracting mechanism. Final cell density, acetate, and ethanol concentrations were similar between these two conditions, but more propionate was consumed (48 ± 12%) than butyrate (31 ± 13%). This indicates that the reduction of carboxylic acids was independent of growth, and that alcohol/aldehyde hydrogenases involved in solventogenesis might have a higher affinity for shorter-chain substrates (i.e., propionate over butyrate). In valerate- and caproate-supplemented cultures, CO consumption was relatively low (34 ± 4% and 23 ± 2%, respectively) even after 28 days of cultivation (vs 9 days for the other conditions), indicating sluggish metabolic activity. The particularly low caproate conversion (1.7 ± 0.7 mM; 10%) can be explained by its higher toxicity compared to shorter carboxylates.^64^ Valerate consumption was similar to that of butyrate (≈5 mM), but the former cultures consumed less CO and produced little biomass (Table 1). This further supports the reasoning that carboxylic acid reduction in *A. wieringae* JM was uncoupled from growth, aligning with observations in the batch bioreactor experiment (Figure 2b,c), and instead served as a mechanism to dispose of reducing equivalents and mitigate carboxylic acid toxicity.

### Cocultivation Modulates the Product Spectrum of *A. wieringae* JM

The impact of cocultivation on the product spectrum of *A. wieringae* JM was investigated in CO-limited chemostats. R1 and R2 underwent two consecutive phases with respective steady states: first, a monoculture of *A. wieringae* JM (in R1 and R2; duplicates), and second, a coculture with either *C. kluyveri* (in R1) or *A. neopropionicum* (in R2). Despite the relatively short HRT applied (48 h), several technical issues throughout the experiment extended the total cultivation time to 140 days. An overview of the fermentation is shown in Figure 3; key bioreactor parameters and steady-state values of each phase are summarized in Table S4.

**Figure 3:**
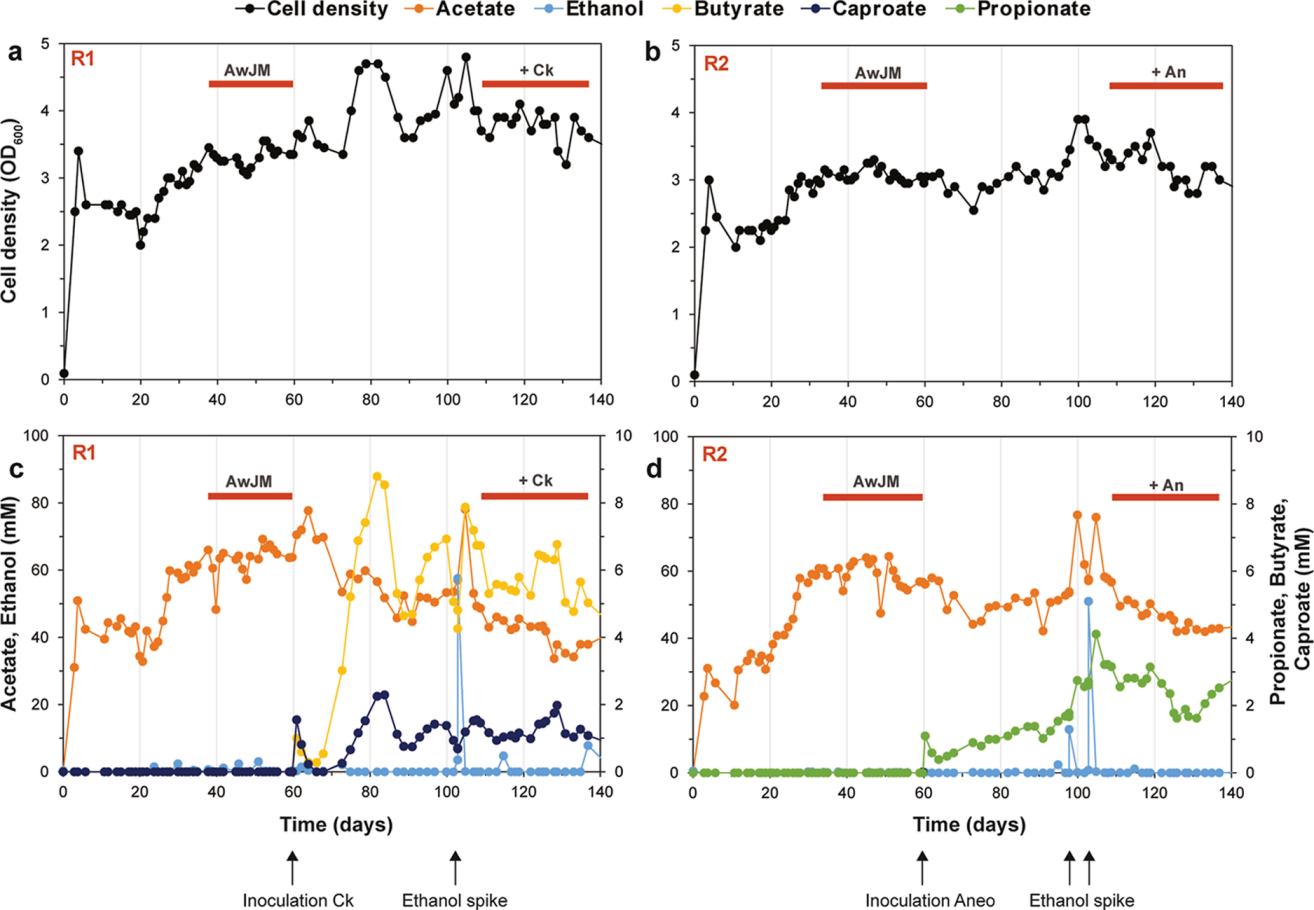
CO fermentation by *A. wieringae* JM in CSTRs in monoculture and in cocultivation with *C. kluyveri* (R1) or *A. neopropionicum* (R2). (a,b) Cell density in R1 and R2, respectively. (c,d) Concentrations of ethanol and carboxylic acids in R1 and R2, respectively. Two phases are distinguished in each bioreactor: monoculture of *A. wieringae* JM (AwJM) (days 0–60), and coculture with *C. kluyveri* (Ck; R1) or *A. neopropionicum* (An; R2) (days 60–140). Horizontal orange bars indicate the steady states of each phase. Arrows denote time points corresponding either to the inoculation of the partner strain or the addition of ethanol to the system (“ethanol spike”), as referred to in the text.

In monoculture, *A. wieringae* JM exclusively produced acetate (63.2 ± 4.6 mM in R1 and 58.7 ± 4.2 mM in R2). During the coculture phases, compared to monoculture, cell density increased by only 5–10% (Figure 3a and Table S4), with acetogenic cells predominating, as confirmed by microscopic observation (not shown). Inoculation of *C. kluyveri* (R1) and *A. neopropionicum* (R2) led to the appearance of their typical products and a decrease in acetate levels, while still dominating (Figure 3c,d). Steady-state concentrations reached were 5.8 ± 0.7 mM butyrate, 1.3 ± 0.3 mM caproate and 41.7 ± 4.5 mM acetate in R1, and 2.4 ± 0.5 mM propionate and 47.1 ± 4.3 mM acetate in R2 (Table S4). Ethanol remained mostly undetected, except for transient accumulations (<3 mM; Figure 3c,d) following stirring peaks of up to 900 rpm caused by system failures (Figure 3)—these disturbances may have triggered overflow metabolism in the acetogen, leading to ethanol production as electron sink.^65^

In both systems, the C3–C6 carboxylic acids could have only been produced from ethanol as electron donor, which was not externally supplied. Thus, while the metabolism of *A. wieringae* JM was entirely acetogenic during monocultivation, cocultivation with the ethanol-consuming species triggered immediate ethanol production. The metabolic shift from acetogenesis to solventogenesis induced by cocultivation was also seen previously in *C. autoethanogenum*.^24,66^ Under conditions of nutrient-limited growth, this shift is thought to be thermodynamically driven rather than regulated by transcriptomic or translational mechanisms.^66–69^ Moreover, a recent study showed that enzymes involved in ethanol production in *A. wieringae* JM are abundant during acetogenesis,^67^ which would facilitate rapid ethanol production when the conversion is rendered favorable. In our experiment, we hypothesize that *C. kluyveri* and *A. neopropionicum* kept ethanol concentrations in the system low enough (<0.1 mM; Table S4) to favor ethanol production by *A. wieringae* JM. Other than ethanol, no alcohols were produced in the chemostat cocultures, unlike in batch cultivation (Figure 2). This can be attributed to the continuous removal of carboxylic acids and the constant supply of fresh medium, preventing conditions necessary for solventogenesis (such as product accumulation to detrimental levels or nutrient limitation). Although increased agitation to enhance CO mass transfer may stimulate higher alcohol production in continuous systems, as reported in other studies,^27,70^ this was not investigated here.

Despite the presence of chain-elongated products, acetate was the dominant product in both bioreactors, indicating limited ethanol production. Ethanol limitation was most noticeable in the coculture with *A. neopropionicum* (R2), where propionate levels increased significantly following ethanol spiking (Figure 3d), but also evident in R1 (Figure 3c). Notably, a significant proportion of the added ethanol was converted to acetate. Ethanol oxidation metabolism has been described in various acetogens such as *C. autoethanogenum*,^24,71^ *A. woodii* ^72^ and *Clostridium ljungdahlii*,^73^ as ethanol may serve as an overflow product of acetogenic metabolism, providing energy storage for conditions of limited substrate availability.^65,71^ Thus, it is likely that *A. wieringae* JM consumed ethanol as an additional electron donor in response to the CO-limited conditions in the chemostats.

The CO consumption rate observed during cocultivation was the same as in the monoculture phase (Table S5), suggesting that, throughout the cultivation, kinetics were dictated by the limited mass-transfer rate of CO into the liquid. CO consumption by *A. wieringae* JM during monoculture was ≈46% (Table S4), significantly lower than observed in CO-limited chemostat cultivation with *C. autoethanogenum* (90%).^66^ However, a lower volumetric CO feeding rate was applied in that system (155 mmol L^–1^ d^–1^ vs ≈430 mmol L^–1^ d^–1^ in this study; Table S4). Consequently, the volumetric CO uptake rate was somewhat higher with *A. wieringae* JM in this study (180–200 mmol L^–1^ d^–1^; Table S4) than with *C. autoethanogenum* (147 mmol L^–1^ d^–1^).^66^ This observation suggests a potentially higher CO utilization efficiency of strain JM, a characteristic that may stem from metabolic adaptations to CO during the extended enrichment preceding its isolation.^36^

### GEM of *A. wieringae* JM and Experimental Validation

Due to the complexity of the system and time constraints, we could not establish the *A. wieringae* JM–*A. neopropionicum*–*C. kluyveri* triculture in continuous bioreactors. As an alternative to experimentally evaluating coculture feasibility and productivity, we constructed the GEM of *A. wieringae* JM, designated AWIEJM-GEM, to subsequently support the development and simulations of a triculture community model. The model of *A. wieringae* JM comprises 623 genes, 1065 metabolites and 1079 reactions, further categorized in Table S5. AWIEJM-GEM is available in multiple formats in our Git repository, ensuring accessibility. The model obtained a MEMOTE score of 73%, indicating good overall quality and reproducibility.

We qualitatively validated the model evaluating growth on 11 carbon sources, including C1 feedstocks and typical heterotrophic substrates (Table 2). The model correctly predicted phenotypes on all the experimentally tested substrates, except methanol, likely due to the incomplete assimilation pathway in the genome annotation. Additionally, the model predicted growth on 1,2-propanediol and alanine, as previously hypothesized based on genome analysis of closely related *Acetobacterium* species.^48^ Quantitative validation of the model was done by comparing predicted acetate and ethanol production rates during growth on CO with experimental data from the chemostat bioreactors. Simulations with flux sampling closely matched the steady-state production rates observed (Table 2). However, while being within the same order of magnitude, the model-predicted acetate production rate was significantly higher (0.65 ± 0.04 mmol h^–1^) than the observed (0.51 ± 0.01 mmol h^–1^). This discrepancy could be due to a slightly higher CO uptake rate used by the model (3.5 ± 0.0 mmol h^–1^) compared to the experimental value (3.3 ± 0.2 mmol h^–1^)—note that CO uptake was constrained within an interval (−3.5 to −3 mmol h^–1^), and the model generated predictions within this range. Alternatively, the differences could be attributed to inaccurate predictions of the ATP maintenance costs (nongrowth-associated maintenance; NGAM). The NGAM reaction (“rxn00062_aw”) of the model was parametrized using experimental values from the duplicate monoculture phases of the chemostats; however, a better fit can be obtained by calculating NGAM and growth-associated maintenance (GAM) from chemostat data at different growth rates, which were not available in this study. Overall, the strong qualitative and quantitative predictions support the reliability of our model of *A. wieringae* JM, AWIEJM-GEM.

**Table 2:** Qualitative and Quantitative Validation of AWIEJM-GEM^a^

	AWIEJM-GEM	experimental
Substrate^b^		
CO	+	+
H_2_ + CO_2_	+	+
formate	+	+
methanol	NA	+
ethanol	+	+
1,2-propanediol	+	NA
glycerol	+	+
lactate	+	+
alanine	+	NA
fructose	+	+
glucose	+	+
Production Rate (mmol h^–1^)^c^		
acetate	0.65 ± 0.04	0.51 ± 0.01
ethanol	0.02 ± 0.02	<−0.01

a Qualitative validation corresponds to growth phenotypes on the listed substrates. Quantitative validation corresponds to steady-state production rates during growth on CO.

b Experimental data from Arantes et al.^36^ +: growth; NA: not available.

c Model-predicted rates were obtained by flux sampling, with growth (0.021 h^–1^) and CO uptake (−3.5 to −3 mmol h^–1^) constrained to experimental values. Experimental data is from the monoculture phase of the two chemostat bioreactors in this study.

### Community GEM of the Tri-culture and Feasibility Study

Following the construction of the GEM of *A. wieringae* JM, we developed a community GEM of the triculture. The model integrates the GEMs of *A. wieringae* JM (AWIEJM-GEM; this study), *A. neopropionicum* (iANEO_607)^34^ and *C. kluyveri* (iCKL708),^34,44^ and is available in our Git repository. We used this model to conduct simulations using cFBA—a suitable approach for modeling continuous cultivation—to assess dilution rates at different biomass ratios that would render the triculture model feasible, thereby informing future experimental design. The feasibility of the model was evaluated across a range of biomass species ratios (1:1:8–8:1:1) and growth rates (0.005–0.08 h^–1^; equivalent to dilution rates in chemostat), with CO as substrate. The resulting solution space is shown in Figure 4.

**Figure 4:**
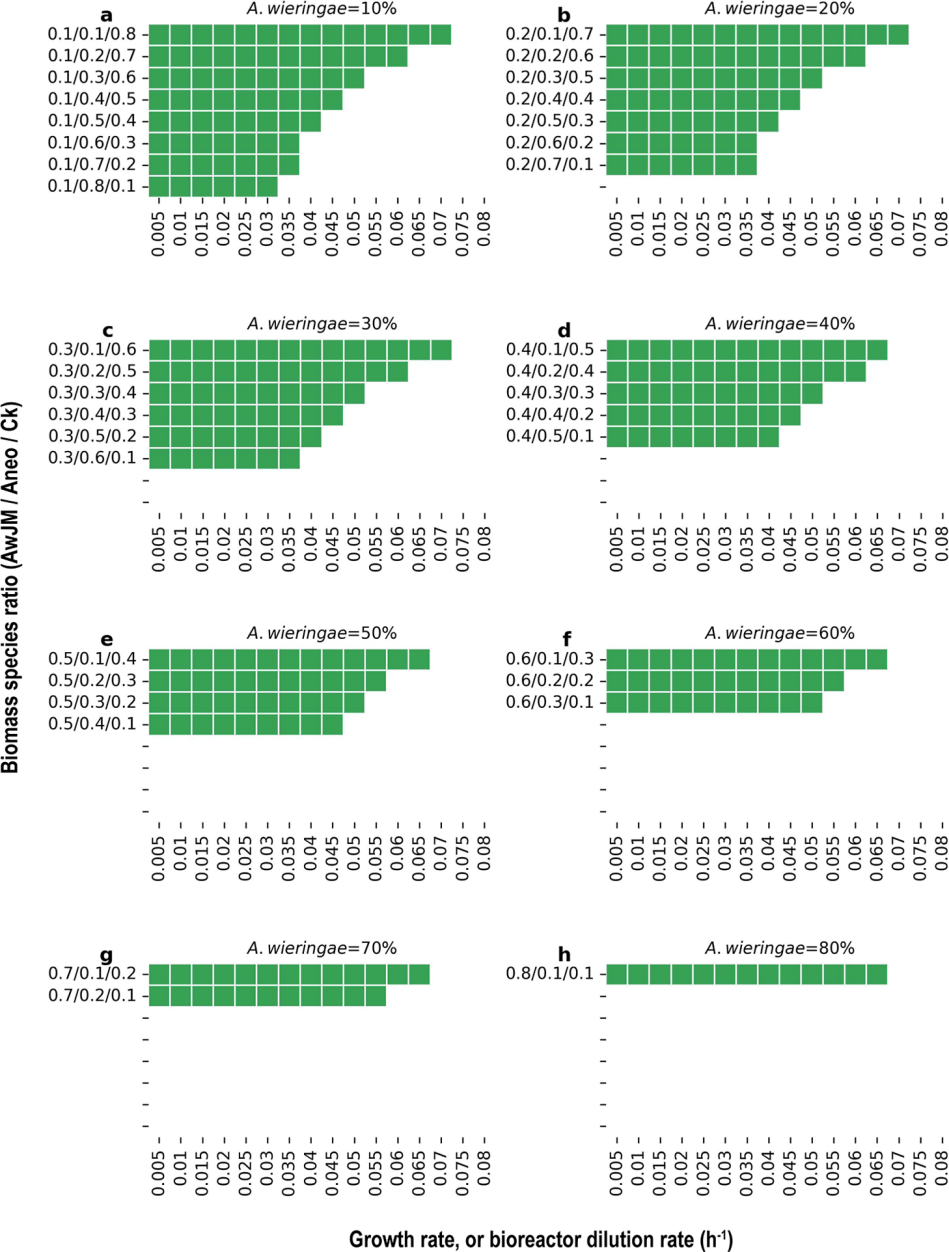
(a–h) Predicted feasible solution space of the triculture model, obtained with cFBA (steady state) for growth on CO at combinations of growth rates (0.005–0.08 h^–1^; *x* axis) and species ratios (0.1–0.8; *y* axis). Each panel corresponds to a fixed abundance of *A. wieringae* JM (10–80%). AwJM: *A. wieringae* JM, Aneo: *A. neopropionicum*, Ck: *C. kluyveri*.

Our analysis shows that, at growth rates up to 0.06–0.07 h^–1^ (HRTs = 16–14 h), the triculture model is feasible when *A. wieringae* JM or *C. kluyveri* are present in either very low or very high abundance while there is a low abundance of *A. neopropionicum*. However, with moderate and high abundances of *A. neopropionicum* (>20%) the triculture model is feasible up to growth rates of 0.05 h^–1^ (HRT = 20 h). Simulation at low and moderate abundance of *A. wieringae* JM (up to 30%) predicted that the triculture was feasible only at growth rates up to 0.03–0.035 h^–1^ (HRTs of 33–28 h). Conversely, despite the three species exhibiting maximum growth rates of 0.1–0.2 h^–1^,^33,71^ simulations at rates exceeding 0.07 h^–1^ (HRT < 33.3 h) predicted the triculture to be unfeasible. The discrepancy may be attributed to the constraints applied to the model parameters; the CO uptake rate and the community biomass were constrained based on experimental data in all conditions, confining the minimum retention time needed to facilitate cross-feeding within the triculture. Simulations at higher CO uptake rates (−5 mmol h^–1^) not shown in the figure, led to a feasible triculture with high abundance of *A. wieringae* JM or*C. kluyveri* even at growth rates of 0.1 h^–1^ due to increased ethanol production (see Git repository). However, it is important to note that simulations were run with a fixed community biomass, and we expect variable community biomass at increased CO uptake rates and growth rates, affecting model predictions.

In our chemostat experiment, conducted at a growth rate of 0.021 h^–1^ (HRT of 48 h), *A. wieringae* JM was predominant in both cocultures, evidenced by the high acetate levels (Figure 3) and microscopic observations (not shown). Therefore, we hypothesize that under those conditions and based on the predicted feasibility of the triculture when *A. wieringae* JM was in high abundance, the acetate accumulated would be sufficient to drive production of ethanol, a crucial intermediate in the triculture, and the establishment of the triculture would have been likely.

### Effect of the Species Ratio on the Product Profile of the Triculture in cFBA Simulations

Given the distinct metabolic capabilities of the three species in the community, we hypothesized that their relative abundances would influence the product profile of the triculture. To explore this and identify conditions that would favor odd-chain production, we performed model simulations of the community model using cFBA with flux sampling at various species ratios. Since *A. wieringae* JM was dominant in our bioreactor experiments, simulations focused on compositions where it constituted the majority (50–80%) of the community, with the relative abundances of *C. kluyveri* and *A. neopropionicum* varying between 10 and 40%. The growth rate (i.e., dilution rate) was fixed at 0.02 h^–1^ to match the conditions used in the coculture chemostat experiments.

Compared to monoculture simulations, ethanol production by *A. wieringae* JM increased markedly in triculture simulations, ranging from 0.08 ± 0.007 to 0.24 ± 0.017 mmol h^–1^ under the tested conditions (Git repository), versus 0.02 ± 0.02 mmol h^–1^ in monoculture (Table 2). The highest production of ethanol predicted coincides when *A. wieringae* JM constituted 80% of the community. This metabolic shift toward solventogenesis induced by cocultivation is consistent with our observations in the chemostat experiment (see Cocultivation Modulates the Product Spectrum of *A. wieringae* JM). Moreover, the simulations showed that ethanol produced by the acetogen was nearly fully consumed by *A. neopropionicum* and *C. kluyveri*, supporting the production of C3–C7 carboxylic acids—again in line with experimental findings (Figure 3). Acetate was the dominant product across nearly all biomass ratios tested (Figure 5a), reaching peak production rates (≈0.2 mmol h^–1^) when *A. neopropionicum* was moderately abundant (30%). This underscores the key role of *A. neopropionicum* in driving acetate production, aligning with previous findings of a near-equivalent propionate-to-acetate production ratio (1.2:1).^34^ In addition, at fixed *A. wieringae* JM abundances (50%, 60% and 70%), increasing the abundance of *A. neopropionicum* led to higher production of propionate (Figure 5a), highlighting this strain’s dependence on ethanol supplied by the acetogen.

**Figure 5:**
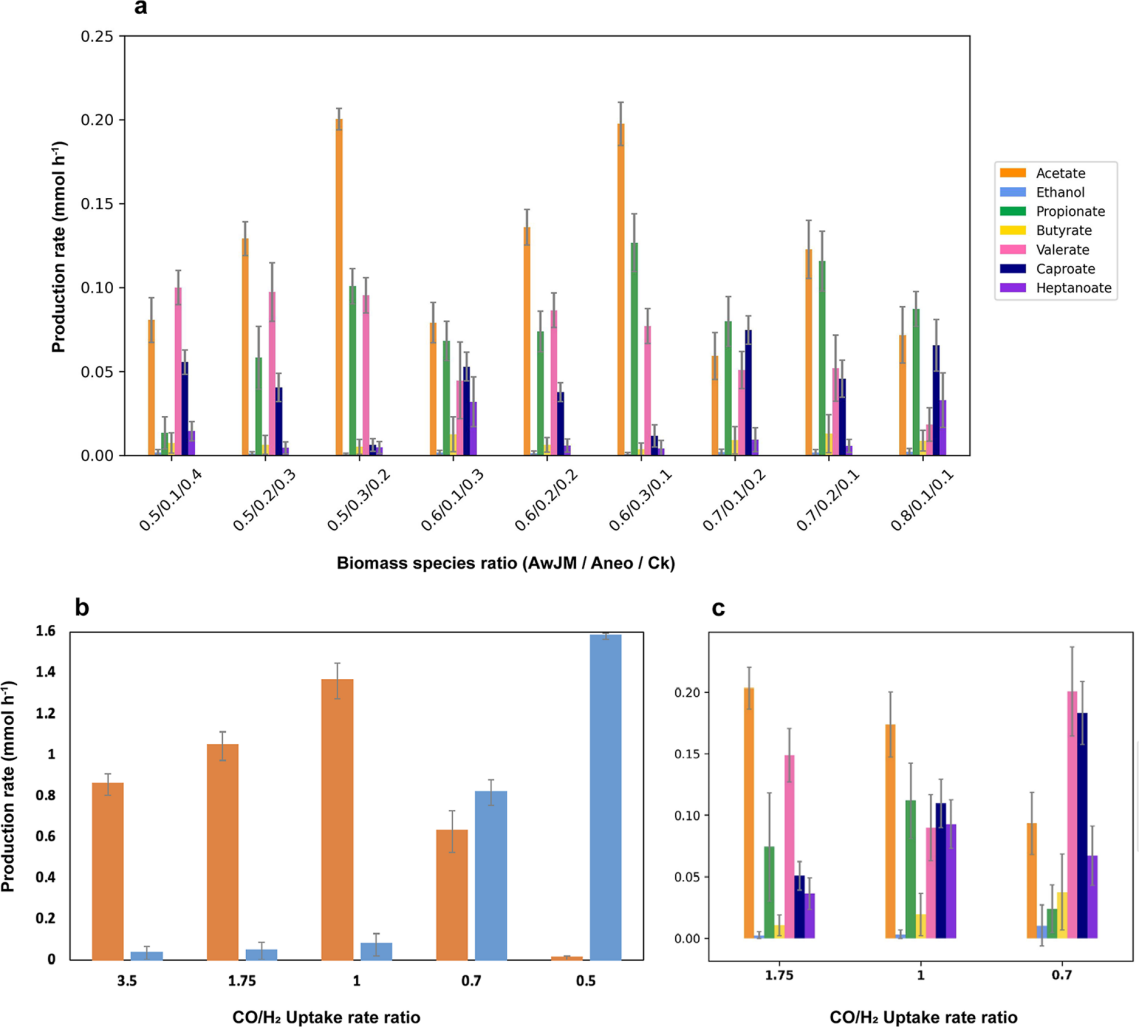
GEM simulations using cFBA and flux sampling. (a) Predicted production rates by the triculture at varying biomass species ratios with *A. wieringae* JM dominant (50–80%). (b,c) Predicted production rates by the *A. wieringae* JM monoculture (b) and the triculture (c), at different CO/H_2_ uptake rate ratios, with a fixed biomass species ratio of 0.5:0.3:0.2 (AwJM/Aneo/Ck). All simulations were performed at fixed growth rate (0.02 h^–1^), total biomass (0.32 g) and CO uptake rate (−3.5 to −2.5 mmol h^–1^), based on experimental data. AwJM: *A. wieringae* JM, Aneo: *A. neopropionicum*, Ck: *C. kluyveri*.

Valerate was predicted to be the dominant MCCA mainly at the lowest abundance of *A. wieringae* JM (50%) (Figure 5a). This suggests that moderate levels of *A. neopropionicum* and *C. kluyveri* (comprising the remaining 50% of the community), combined with robust acetogenic activity to support CO conversion to ethanol, are key to enabling propionate availability and chain elongation activity—both essential for valerate accumulation. In contrast, higher abundances of *A. wieringae* JM combined with low *A. neopropionicum* levels (10%) favored caproate over valerate, a shift that can be attributed to limited propionate availability, as reflected in the model-predicted fluxes (Git repository). Heptanoate production was highest at species ratios of 0.6:0.1:0.3 and 0.8:0.1:0.1 (AwJM/Aneo/Ck), where increased ethanol availability to *C. kluyveri* (reflected in the model-predicted fluxes; Git repository) stimulated chain elongation toward more reduced products, a pattern consistent with previous chain elongation studies.^22,53^ Overall, simulations indicated that valerate production was maximized when *A. wieringae* JM comprised 50–60% of the community, with balanced proportions (20–30%) of both *A. neopropionicum* and *C. kluyveri*. On the other hand, low *A. neopropionicum* abundances favored heptanoate formation but coincided with increased caproate, as expected from enhanced chain elongation by *C. kluyveri*.

The interdependencies inferred from the simulations—such as the reliance of *A. neopropionicum* on ethanol from *A. wieringae* JM—are consistent with observations in the chemostat cocultures (Figure 3), supporting the predictive value of cFBA in capturing key microbial interactions to simulate continuous operations. However, the model predictions do exhibit a degree of error—particularly high for heptanoate, butyrate and other products in certain scenarios (Figure 5a)—reflecting inherent model limitations and the need for further refinement to improve predictive accuracy. For example, in the *A. wieringae* JM–*C. kluyveri* chemostat cocultivation (with dominance of the acetogen and at the same dilution rate as the model simulations; 0.02 h^–1^) butyrate production exceeded that of caproate (Figure 3c), suggesting a lower ethanol/acetate ratio than predicted under similar conditions. Importantly, ethanol uptake by the acetogen under CO limitation, observed in the chemostat experiment (see Cocultivation Modulates the Product Spectrum of *A. wieringae* JM), is not captured by the model, as ethanol was only defined as product. The model also predicted faster consumption of propionate than acetate by *C. kluyveri*, but enzyme kinetics were not included, potentially affecting accuracy. Addressing these limitations with kinetic models,^74^ thermodynamic flux analysis (TFA),^75^ or metabolism-expression (ME) models^76^ that integrate proteome-cost constraints, could help resolve these limitations and better explain the observed metabolic shifts during cocultivation.

### Effect of H_2_ Supplementation on Product Profiles in cFBA Simulations

Given the relevance of H_2_ in syngas fermentation, serving as additional electron donor to mitigate carbon loss,^77^ we performed cFBA simulations with flux sampling at a range of CO/H_2_ uptake ratios (3.5–0.5) to evaluate its effect on the product profile of *A. wieringae* JM (monoculture). Under high ratios (3.5, 1.75, and 1), acetate was the dominant product, with production increasing as the ratio decreased, reaching a maximum (1.33 ± 0.12 mmol h^–1^) at a ratio of 1 (Figure 5b). Ethanol production remained low across these conditions (<0.1 mmol h^–1^), and only slightly higher than the observed rate in chemostat (steady state) without H_2_ present (Table 2). As the CO/H_2_ uptake ratio further decreased (0.7 and 0.5), both acetate and ethanol production increased, with ethanol becoming the dominant product at the lowest CO/H_2_ ratio. At a CO/H_2_ ratio of 0.5, ethanol reached its highest production rate (1.58 ± 0.01 mmol h^–1^), while acetate production was nearly eliminated (Figure 5b). This observed shift in product distribution indicates that H_2_ supplementation promotes ethanol formation in acetogen, aligning with previous findings.^69^ The overall increase in product formation with higher H_2_ availability also suggests enhanced carbon fixation due to the presence of an additional electron donor—H_2_—facilitating the reduction of CO_2_. According to theoretical stoichiometry of CO/H_2_ conversion, no carbon loss as CO_2_ is expected at a CO/H_2_ uptake rate of 0.5 (or H_2_/CO uptake of 2),^77^ which aligns with our model predictions, as CO_2_ production was zero at this condition (Git repository). In our simulations, we observed a 70% higher carbon flux through the Wood–Ljungdahl pathway and 100% and 94% lower CO_2_ production with CO/H_2_ uptake rate ratios of 0.5 and 0.7, respectively, compared to 3.5 (Git repository).

The effect of H_2_ supplementation was also evaluated on the triculture model at CO/H_2_ uptake ratios of 1.75, 1 and 0.7, for a fixed biomass species ratio of 0.5:0.3:0.2 (*A. wieringae* JM/*A. neopropionicum*/*C. kluyveri*). We chose this biomass ratio as it produced a feasible model outcome (Figure 4) with acetogen dominance, consistent with experimental observations. Our simulations suggest that increasing H_2_ availability in the triculture decreases acetate accumulation in favor of other carboxylic acids (Figure 5c). In addition, the production rates of odd-chain products (C5 + C7) peaked (0.279 ± 0.043 mmol h^–1^) at the highest CO/H_2_ ratio (0.7), with valerate as the dominant MCCA, followed by caproate and heptanoate. Similarly to monoculture simulations, here we observed that H_2_ availability stimulates ethanol production (up to 0.83 mmol h^–1^; Git repository) by enhancing carbon fixation, as evidenced by a drop in CO_2_ output (<0.1 mmol h^–1^; Git repository). This, in turn, promotes propionigenesis and chain elongation, favoring odd-chain product formation. Interestingly, simulations performed at a biomass species ratio of 0.7:0.1:0.2 and CO/H_2_ uptake ratio of 0.7 (Git repository) predicted heptanoate as the most abundant product, with the highest rate observed in our simulations (0.16 ± 0.017 mmol h^–1^). While these simulations offer insights into the role of H_2_ in product distribution, it should be noted that the model does not account for potential inhibitory effects such as CO-induced hydrogenase inhibition or product accumulation toxicity, which may impact actual system performance.

### Unlocking the Potential of Synthetic Cocultures for Syngas Fermentation

In this study, we demonstrated that the strategic selection and combination of microbial strains in a defined coculture enables the selective (though not exclusive) production of C5 compounds from CO/CO_2_, products rarely observed in syngas fermentation. The accumulation of 6 mM valerate (0.61 g L^–1^) and 3.8 mM pentanol (0.33 g L^–1^) obtained here (Figure 2) are, to our knowledge, the highest reported concentrations of these products from CO/syngas, and superior than obtained with open mixed culture.^32^ However, the low specificities (mol product/mol total products) highlight the need for optimization strategies. The coculture described in this work thus represents a foundation for further development that, as shown here, can be guided by genome-scale metabolic modeling. Our model of *A. wieringae* JM (AWIEJM-GEM) fitted well the experimental phenotypes (Table 2), underscoring its reliability. The triculture GEM captured all key metabolic exchanges (as depicted in Figure 1), and provided insights into intracellular fluxes and patterns observed under chemostat cultivation. Besides the aforementioned limitations, an aspect to be addressed in future community modeling efforts is the incorporation of observed population abundances,^42^ which can strongly influence the product profile but are often not reported in experimental studies. In our simulations, we observed that biomass species ratios, together with the dilution rate, determined the feasibility of the triculture model (Figure 4) and modulated product selectivity (Figure 5a). Microbial community engineering can be achieved, for example, by imposing spatiotemporal separation, applying directed evolution, or adjusting parameters such as pH, temperature or dilution rate to favor one strain over others.^78,79^ Our cFBA simulations further underscored the advantage of cofeeding H_2_ with CO to enhance carbon fixation, resulting in increased ethanol production by *A. wieringae* JM (Figure 5b). In triculture simulations, this boosted propionate production and chain elongation, thereby enhancing valerate and heptanoate formation (Figure 5b). However, H_2_ supplementation targeting valerate (e.g., with a species ratio of 0.5:0.3:0.2) or heptanoate (e.g., 0.7:0.1:0.2) also resulted in increased formation of even-chain products. Future work should focus on experimentally validating these predictions and optimizing gas composition—alongside with parameters such as dilution rate—for targeted MCCA production. Further improvement in odd-chain selectivity could be achieved by increasing propionate availability, either through direct supplementation^80^ or by promoting propionate-producing species within the community, and by controlling the ethanol-to-propionate ratio.^53^ Limiting acetate accumulation in favor of ethanol, for example by downregulating acetogenesis through metabolic engineering^81^ or adaptive laboratory evolution,^82^ may also help steer chain elongation toward odd-chain products.^53^ Two-stage processes, where acidogenesis and solventogenesis efficiently take place in separate bioreactors, are likely more advantageous for alcohol production (over MCCAs) from syngas.^27,60,83^ Finally, given the heterogeneous composition and potential impurities of syngas,^84^ future studies should also prioritize experiments using real syngas to evaluate its impact on microbial interactions and process performance.

## Supplementary Material



## Data Availability

The genome-scale metabolic model of *A. wieringae* JM, AWIEJM-GEM, and the data generated by the model simulations are available in the following Gitlab repositories: https://gitlab.com/wurssb/Modelling/AWIEJM-GEM and https://gitlab.com/wurssb/Modelling/triculture_aw_an_ck.

## Notes

Supporting Materials and Methods: detailed protocol of the reconstruction and validation of the GEM of *A. wieringae* JM (AWIEJM-GEM); representation of the construction of the community GEM of the *A. wieringae* JM–*C. kluyveri*–*A. neopropionicum* triculture, and the mathematical equations and assumptions of the triculture model simulations using cFBA. Supporting Figures: microscopy images of the triculture during batch bioreactor fermentation (Figure S1); partial pressures of gases during the batch bioreactor fermentation (Figure S2); scheme of the construction of the triculture GEM (Figure S3). Supporting Tables: composition of the trace element solution from DSMZ medium 318 (Table S1), of the vitamins solution (Table S2), and of the modified trace element solution from medium ATCC 1754 (Table S3); steady-state values of the chemostat cultivations of *A. wieringae* JM with *C. kluyveri* and *A. neopropionicum* (Table S4); composition of the AWIEJM-GEM (Table S5) (PDF)

## ∥

Genome Biology Unit, European Molecular Biology Laboratory, 69117 Heidelberg, Germany

## ⊥

Galactic S.A., 1070 Brussels, Belgium.

## #

Bioprocess Engineering, Wageningen University & Research, 6708PB Wageningen, The Netherlands.
