# Supplementary material for: Synthetic Microbial Cocultivation for Targeted Production of Odd-Chain Carboxylates and Alcohols from Carbon Monoxide
Source: Environ Sci Technol. 2025 Aug 27;59(35):18706–21. doi: 10.1021/acs.est.4c14794 (PMC12424164; doi:10.1021/acs.est.4c14794)
Supplement: Supplementary file 1 [file es4c14794_si_001.pdf]

Supporting Information

Synthetic Microbial Co-cultivation for Targeted Production of Odd-Chain Carboxylates and Alcohols from Carbon Monoxide

Ivette Parera Olm, Sara Benito-Vaquerizo, Charles Dubaere, Vitor A.P. Martins dos Santos, Maria Suarez-Diez, Diana Z. Sousa

Supporting Materials and Methods

Figures S1–S3

Tables S1–S5

Supporting References

SUPPORTING MATERIALS AND METHODS

Construction of the GEM of *Acetobacterium wieringae* JM (AWIEJM-GEM). The genome-scale metabolic model (GEM) of *A. wieringae* JM was reconstructed using the GEM of *Clostridium autoethanogenum* (iCLAU786)[1] as scaffold and subsequently adapted according to the genomic features of *A. wieringae* JM following an orthology-based approach. First, the genomic sequence of *A. wieringae* JM (GCA_008107585.1)[2] and the reference annotation were retrieved in GFF format from the National Center for Biotechnology Information (NCBI). The genome was functionally annotated using eggNOG-mapper 2.1.7[3] and structurally annotated using the reference annotation; the annotation can be found in the public GitLab repository: https://gitlab.com/wurssb/Modelling/AWIEJM-GEM. The genomic sequence of *C. autoethanogenum* DSM 10061^T (GCA_001484725.1)[4] was retrieved from NCBI in FASTA format, and OrthoFinder 2.5.4[5] was used to identify orthologous genes between the two species. Then, the scaffold model of *C. autoethanogenum* (iCLAU786)[1] was modified for *A. wieringae* JM. Reactions with gene-protein-reaction (GPR) associations of genes with predicted orthologs in *A. wieringae* JM were kept. A new GPR identifier was associated to these reactions by replacing the *C. autoethanogenum* locus tag ('CAETGH_RSXXXXX') by the corresponding *A. wieringae* JM protein ID 'TYCXXXXX'. Reactions for which no homologous genes had been found were further inspected. Enzyme Commission (EC) numbers describing these reactions were retrieved from the template model, and the gene(s) associated to these EC numbers in *A. wieringae* JM were retrieved either from the genome annotation file or from the PATRIC[6] or UniProt[7] databases. Reactions without an annotated EC number in the template model were searched by their corresponding identifiers in either ModelSEED,[8] KEGG,[9] MetaCyc[10] or BIGG[11] databases. The EC numbers obtained were used to retrieve the corresponding genes from either the genome annotation file, PATRIC or UniProt databases. The stoichiometry and mass balances of the reactions were verified in

the aforementioned databases. The biomass reaction was kept the same as in the model of *C. autoethanogenum*. The draft model was transformed into SBML (xml) format using Python and COBRApy.[12] Next, flux balance analysis (FBA) within COBRApy was used to maximize growth on CO and on H_2/CO_2. The uptake rates of CO, H_2 and CO_2 were fixed at 30 mmol g_{CDW}^{-1} h^{-1}, 40 mmol g_{CDW}^{-1} h^{-1} and 20 mmol g_{CDW}^{-1} h^{-1}, respectively. Orphan reactions — those without an associated GPR —, were removed, with exception of extracellular and transport reactions. We also checked whether the growth rate and the acetate production rate under CO and H_2/CO_2 growth were affected if each reaction from the template model of *C. autoethanogenum* were removed. Reactions from the template model that led to the same rates when removed were excluded from the model as they were considered not essential and/or not present in *A. wieringae* JM. To eliminate these reactions, the function single_reaction_deletionReactions of COBRApy was used. Metabolites involved exclusively in the removed reactions were also eliminated. The model was amended with reactions and metabolites whose associated proteins were annotated in the genome of *A. wieringae* JM and reported to be present in other *Acetobacterium* species[13] but not in *Clostridium*. Additionally, the GEM of the close relative *Acetobacterium woodii* ('CNA_AW')[14,15] was used to refine the model. Reactions were added by keeping the same information and namespace of the scaffold model using ModelSEED. Once new reactions were added, the previous procedure was repeated to identify possible non-essential orphan reactions. The final model in format xml was translated into SBML Level 3 Version 1 using KBase.[16] The model was validated using MEMOTE[17] and SBML Validator.[18] The final GEM of *A. wieringae* JM, AWIEJM-GEM, can be found in the git repository and in BioModels (MODEL2310100001) in Table format, json, yaml, matlab and SBML L3V1 standardization, together with a MEMOTE report of quality assessment. The model composition is shown in Table S5.

Qualitative Validation of AWIEJM-GEM. The model was qualitatively validated by assessing growth phenotypes on carbon sources that had been tested in *A. wieringae* JM, *A. wieringae*^{T} or other *Acetobacterium* species,[2,13] or that had been predicted as substrates by the model (Table 1). Simulations were done using FBA in COBRApy version 0.24.0 and Python 3.9. For each carbon source, the lower bound of the substrate uptake rate per time point was constrained to -30 mmol g_{CDW}^{-1} h^{-1} when assessing growth on a single carbon source, and to -30 mmol g_{CDW}^{-1} h^{-1} in total when assessing growth on more than one carbon source, unless specified otherwise. The biomass synthesis reaction was used as the objective function. Growth was considered positive when the growth rate was higher than 0.0001 h^{-1}.

Quantitative Validation of AWIEJM-GEM. Quantitative validation of the model was done by comparing acetate and ethanol production rates predicted by the model with those determined experimentally in chemostat cultivations of *A. wieringae* with CO as substrate (this study). Rates were expressed as environmental fluxes (mmol h^{-1}) instead of specific fluxes (mmol g_{CDW} h^{-1}), as previously done[19]. The lower bound of the CO uptake rate was constrained between -3.5 mmol h^{-1} and -1 mmol h^{-1}. The growth rate was constrained to 0.021 h^{-1}. The biomass reaction was constrained by the growth rate multiplied by the measured amount of biomass in the bioreactor during steady state. The non-growth associated maintenance (NGAM) of the ATP maintenance reaction ('rxn00062_aw') was adjusted based on the CO consumed in the chemostat cultivation experiment, and it was used to constrain the lower bound of this reaction. The solution space and the set of fluxes compatible with the measured constraints were sampled using the sample function in the flux_analysis submodule of COBRApy. The results presented are the average and standard deviation of 10000 iterations.[20–23]

Simulations of the Community GEM using cFBA: Modeling framework

The community modeling framework followed in this study is based on community FBA and SteadyCom[24]. The approach considers steady state and balanced growth of the microbial species in the community.

In traditional FBA, fluxes are computed maximizing the biomass reaction as follows:

Max v^{s}_{biomass}

$v^{s}_{\text{biomass}} = \mu$; growth rate of species s in h^{-1}

Subject to: $\Sigma\, S_{mr}^{s}\, v_r^{s} = 0,\ \forall\ m \in M^{s}$ (Eq. 1)

$LB_r^{s} \leq v_r^{s} \leq UB_r^{s}\ \ \forall\ r \in R^{s}$ (Eq. 2)

v_r^{s} ; Specific flux of reaction r (mmol g_{DW}^{-1} h^{-1})

S_{mr}^{s} ; Stoichiometry of metabolite m in reaction r

LB_r^{s}, UB_r^{s} ; lower and upper bounds of v_r^{s} fluxes

M^{s}, R^{s}; set of metabolites and reactions for species s, respectively.

In single-species models, all fluxes are relative to the abundance of the represented species. However, in a community model, each species may have a different abundance. In order to input the abundance of each species correctly, we work with aggregate fluxes (V_r^{s}; mmol h^{-1}), and balance the biomass reactions accordingly:

$V_r^{s} = X^{s}\, v_r^{s}\ ,\ \forall\ r \in R^{s},\ s \in S$

$X^{s} = X^{T}\, \phi^{s}$

X^{T}; Total community biomass in g

ϕ^{s} : biomass species ratio

X^{s}; biomass of species s in g

V_r^{s}; Environmental flux or aggregate flux of reaction r in species s (mmol h^{-1})

R, S; all reactions of species s and all species in the community, respectively.

Mass-balance equation in the community (M^{c}):

$u_m^c - p_m^c + \sum_{s \in \mathbf{S}} V^s_{ex(m)} = 0, \forall\ m \in M^c$

u_m^c ; uptake rate of metabolite *m* in the community/ Environmental flux (mmol h^{-1})

p_m^c ; production rate of metabolite *m* in the community /Environmental flux (mmol h^{-1})

$V^s_{ex(m)}$; Transport reaction fluxes from the extracellular compartment to the intracellular compartment of species s.

Now, Equation 1 and Equation 2 are arranged as follows:

$\sum S_{mr}^s V_r^s = 0, \forall\ m \in M^s$ (Eq. 1)

$LB_r^s X^s \leq V_r^s \leq UB_r^s X^s \quad \forall\ r \in R^s,\ s \in S$ (Eq. 2)

Finally, we input the biomass species ratio as stoichiometric composition of each species biomass contribution in the community biomass reaction ($V^c_{biomass}$), and solve the solution space as follows:

Max V^c_{biomass}

with $V^c_{\boldsymbol{biomass}} = X^T \mu = \sum V^s_{biomass} = \sum X^T \phi^s \mu \ \ \forall\ s \in \mathbf{S}$

When specified, we used flux sampling to sample the solution space. In this instance, there is no need to define an objective function, and fluxes are computed under the specified constraints.

SUPPORTING FIGURES

a

b

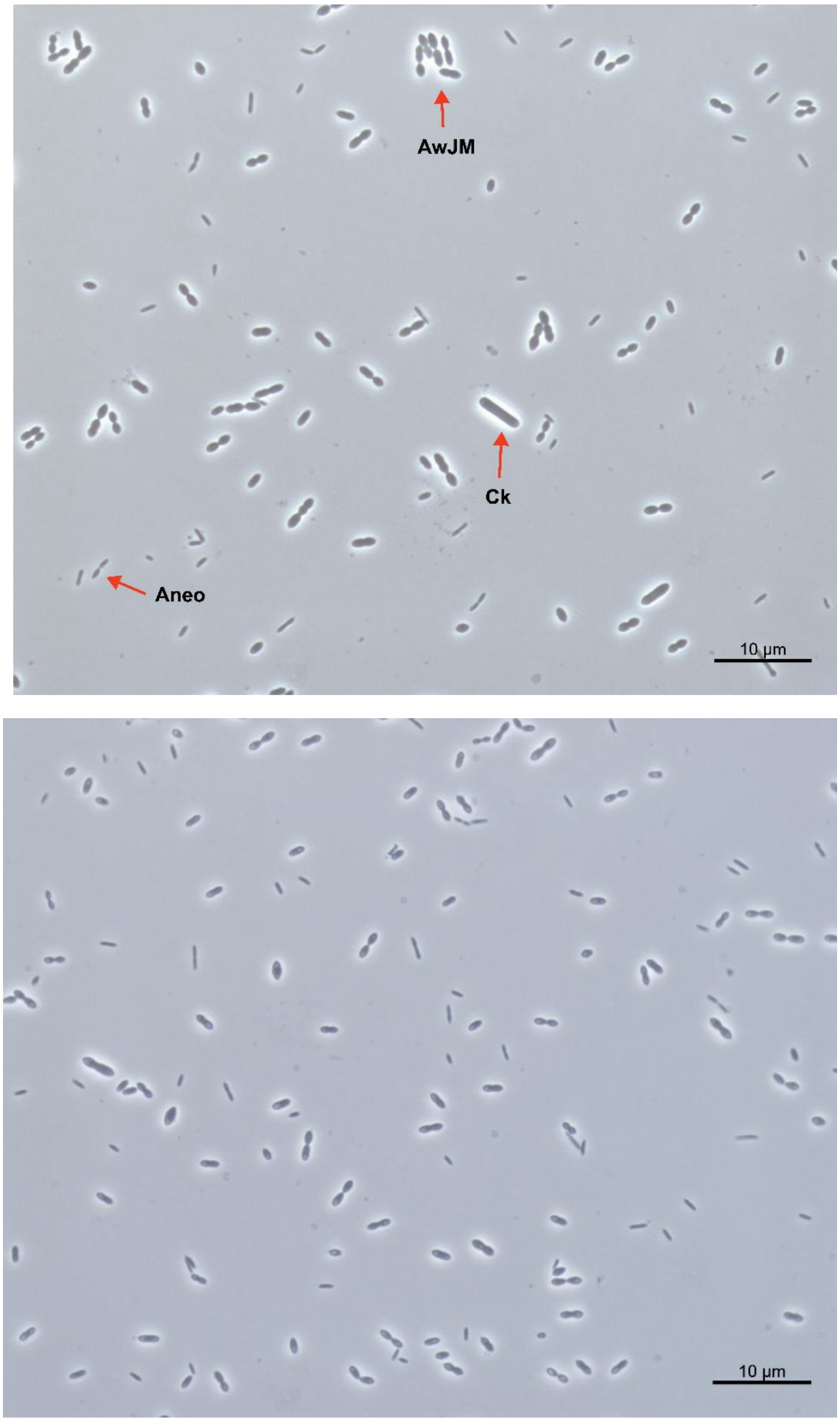

Figure S1. Phase-contrast microscopy images of the synthetic tri-culture during batch bioreactor fermentation. Samples taken during the growth phase at (**a**) 86 h and (**b**) 110 h. AwJM: *A. wieringae* JM, Ck: *C. kluyveri*, Aneo: *A. neopropionicum*.

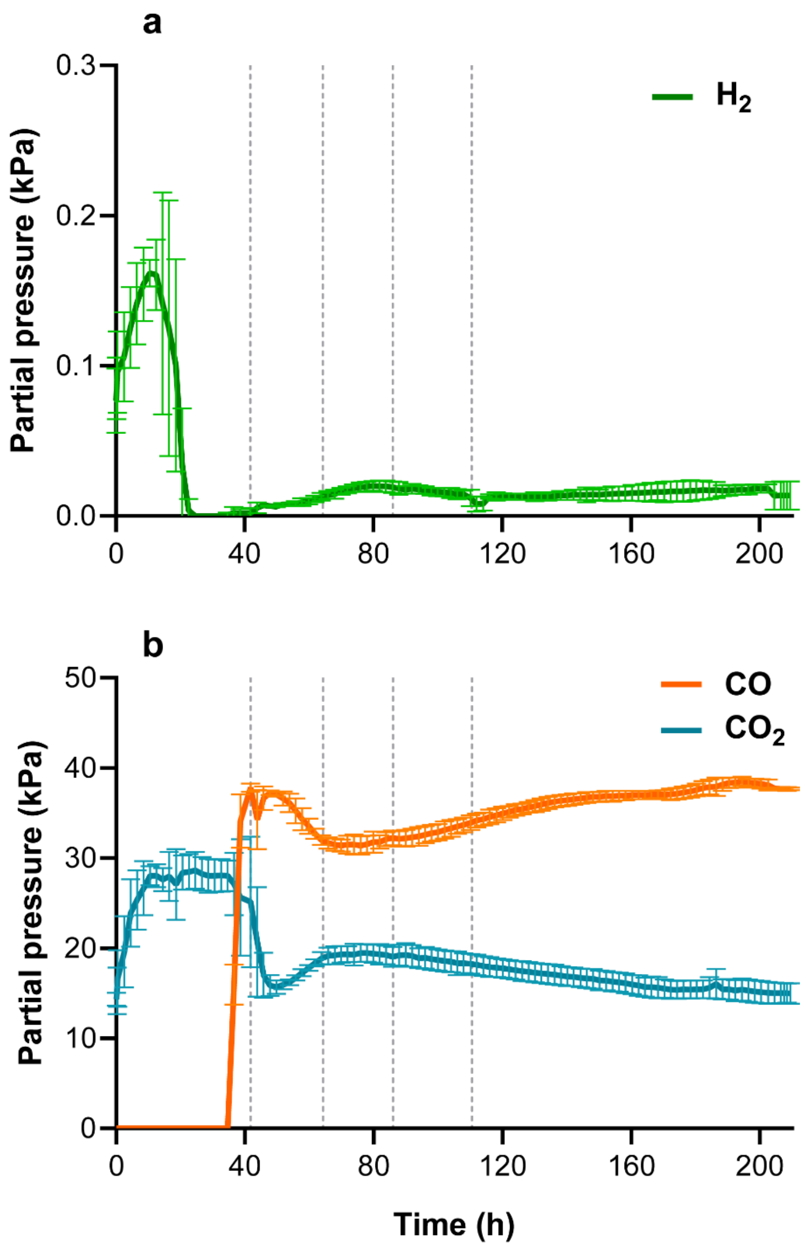

Figure S2. Headspace partial pressures of H_2 (**a**), CO and CO_2 (**b**) in the batch bioreactor fermentation with the synthetic tri-culture. Values shown are the averages of quadruplicate bioreactors, with error bars representing standard deviations. N_2 was present as makeup gas (not shown). The first dashed line corresponds to the inoculation of *A. wieringae* JM and start of the CO inflow. The subsequent dashed lines indicate gradual increases of the agitation from 100 rpm to 150, 250 and 300 rpm.

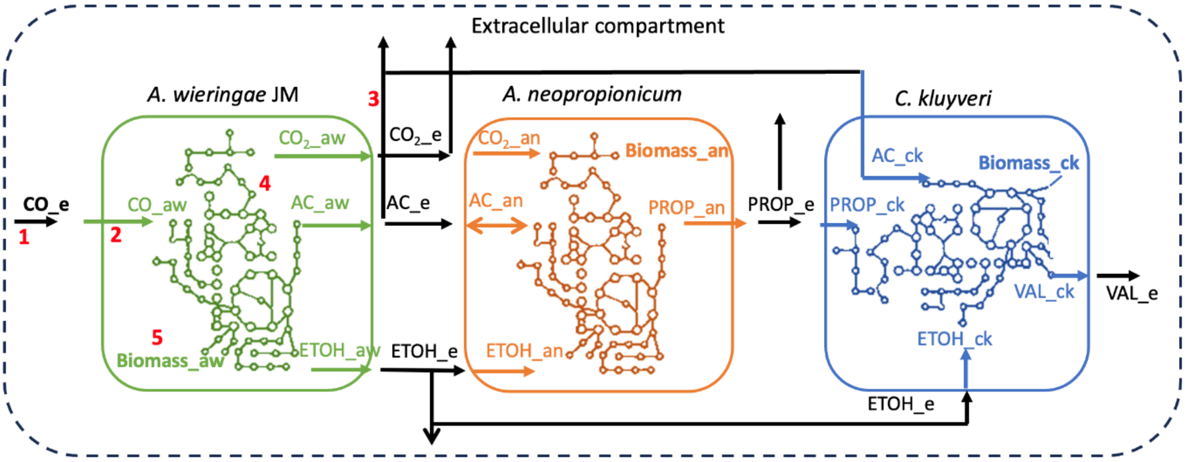

1: CO_e : CO uptake rate / Exchange reaction/Environmental flux
2: CO_e → CO_aw : CO transport reaction from the extracellular compartment to the intracellular compartment of *A. wieringae*
3: AC_e → : Acetate production rate /Exchange reaction
4. X_aw + Y_aw → Z_aw : Intracellular reaction in *A. wieringae* model ; X, Y , Z : Intracellular metabolites
5: Biomass components → Biomass_aw : Biomass reaction of *A. wieringae*
ϕ^{aw}Biomass_aw + ϕ^{an}Biomass_an + ϕ^{ck}Biomass_ck → Biomass_e : Community biomass reaction ; ϕ^{s}: biomass species ratios

Figure S3. Schematic representation of the construction of the tri-culture community GEM. Each colored squared represents the model of an individual species, designated as intracellular compartments. The dashed line frames the shared extracellular compartment. Metabolites within the extracellular space are classified as extracellular, while those within individual species models are intracellular. Arrows indicate reaction directions within the model. Numbers in red (1–5) correspond to different reaction types present in the tri-culture model, as detailed in the figure legend. The schematic highlights only some metabolites and interactions; for a comprehensive overview of all metabolic fluxes, refer to the main text and the Git repository.

SUPPORTING TABLES

Table S1. Composition of the trace element solution from DSMZ medium 318

**Component**	**Concentration ($g L^{-1}$)**
Nitriloacetic acid (NTA)	12.8
$FeCl_2$ x 4 H_2O	1.0
$MnCl_2$ x 4 H_2O	0.1
$CoCl_2$ x 6 H_2O	0.03
$CaCl_2$ x 2 H_2O	0.1
$ZnCl_2$	0.1
$CuCl_2$	0.02
H_3BO_3	0.01
Na_2MoO_4 x 2 H_2O	0.03
$NiCl_2$ x 6 H_2O	0.1
NaCl	1.0
Na_2SeO_3 x 5 H_2O	0.03
Na_2WO_4 x 2 H_2O	0.04

Table S2. Composition of the vitamins solution

**Component**	**Concentration ($mg L^{-1}$)**
p-Aminobenzoate (PABA)	100
Riboflavin (B2)	100
Thiamin (B1)	200
Nicotinamide/Niacin/Nicotinate (B3)	200
Pyridoxin (B6)	500
Pantothenate (B5)	100
Cobalamin (B12)	100
Biotin (B7)	20
Folate (B9)	50
Thioctic acid	50

Table S3. Composition of the modified trace element solution from medium ATCC 1754

Component	Amount per liter	
Nitriloacetic acid (NTA)	2.0	g
$MnSO_4$ x H_2O	1.0	g
$Fe(SO_4)_2(NH_4)_2$ x 6 H_2O	0.8	g
$CoCl_2$ x 6 H_2O	0.2	g
$ZnSO_4$ x 7 H_2O	0.2	mg
$CuCl_2$ x 2 H_2O	20.0	mg
$NiCl_2$ x 6 H_2O	20.0	mg
Na_2MoO_4 x 2 H_2O	20.0	mg
Na_2SeO_4	20.0	mg
Na_2WO_4	20.0	mg

Table S4. Steady-State Parameters of *A. wieringae* JM Chemostat Bioreactors[a]

	R1		R2	
**Parameter**	**Monoculture**	**Co-culture**	**Monoculture**	**Co-culture**
CO volumetric inflow rate (mmol L^{-1} d^{-1})	424		434	
HRT (h)	48.1		46.6	
D (h^{-1})	0.021		0.021	
**Steady-state period (days)**	**38 - 60**	**109 – 137**	**34 - 60**	**109 – 137**
Headspace pCO (kPa)	51.7±1.3	47.7±2.2	53.4± 2	52.4±2.7
Headspace pH_2 (kPa)	0.07±0.01	0.06±0.01	0.06±0.01	0.04±0.01
Headspace pCO_2 (kPa)	12.4±1.2	14.5±2.2	11.5±1.3	11.1±2.1
CO vol. uptake rate (mmol L^{-1} d^{-1})	202±12	215±16	188±23	188±16
CO utilization (%)	48±3	51±4	44±5	43±4
Biomass (g L^{-1})	0.80±0.06	0.89±0.03	0.79±0.11	0.85±0.03
Acetate (mM)	63.2±4.6	41.7±4.5	58.7±4.2	47.1±4.3
Ethanol (mM)	0.51±0.87 [a]	ND	<0.02	<0.01
Propionate (mM)	ND	ND	ND	2.4±0.5
Butyrate (mM)	ND	5.8±0.7	ND	<0.01
Caproate (mM)	ND	1.3±0.3	ND	ND
Acetate vol. production rate (mmol L^{-1} d^{-1})	31.1±2.3	20.5±2.2	29.9±2.1	24.0±2.2
Ethanol vol. production rate (mmol L^{-1} d^{-1})	0.25±0.43[b]	0.6±0.1	<0.09	<0.02

[a]Values shown are the average and standard deviation of several measurements during steady state. R1 and R2 correspond to the bioreactor with *C. kluyveri* and *A. neopropionicum*, respectively, during the co-culture phase. HRT: hydraulic retention time; D: dilution rate; ND: not detected. [b]The large standard deviations are due to transient ethanol accumulation (<3 mM) caused by sporadic system failures.

Table S5. Composition of the model AWIEJM-GEM

Features	Abundance
**Genes**	623
**Metabolites**	1065
Intracellular metabolites	914
Extracellular metabolites	151
**Reactions**	1079
Conversion reactions	906
Transport reactions	98
Exchange reactions	75
**Reactions associated with genes**	773 (71.6%)
**Reactions non-associated with genes**	306 (28.4%)

SUPPORTING REFERENCES

1. Valgepea, K. *et al.* Arginine deiminase pathway provides ATP and boosts growth of the gas-fermenting acetogen *Clostridium autoethanogenum*. *Metabolic Engineering* **41**, 202–211 (2017).
2. Arantes, A. L. *et al.* Enrichment of anaerobic syngas-converting communities and isolation of a novel carboxydotrophic *Acetobacterium wieringae* strain JM. *Frontiers in Microbiology* **11**, 58 (2020).
3. Huerta-Cepas, J. *et al.* Fast genome-wide functional annotation through orthology assignment by eggNOG-mapper. *Molecular Biology and Evolution* **34**, 2115–2122 (2017).
4. Humphreys, C. M. *et al.* Whole genome sequence and manual annotation of Clostridium autoethanogenum, an industrially relevant bacterium. *BMC Genomics* **16**, (2015).
5. Emms, D. M. & Kelly, S. OrthoFinder: phylogenetic orthology inference for comparative genomics. *Genome Biology* **20**, (2019).
6. Davis, J. J. *et al.* The PATRIC Bioinformatics Resource Center: expanding data and analysis capabilities. *Nucleic Acids Research* **48**, D606–D612 (2019).
7. UniProt Consortium, T. UniProt: the universal protein knowledgebase. *Nucleic Acids Research* **46**, 2699 (2018).
8. Henry, C. S. *et al.* High-throughput generation, optimization and analysis of genome-scale metabolic models. *Nat Biotechnol* **28**, 977–982 (2010).
9. Ogata, H. *et al.* KEGG: Kyoto Encyclopedia of Genes and Genomes. *Nucleic Acids Research* **27**, 29–34 (1999).
10. Karp, P. D., Riley, M., Paley, S. M. & Pellegrini-Toole, A. The MetaCyc database. *Nucleic Acids Research* **30**, 59–61 (2002).

11. Schellenberger, J., Park, J. O., Conrad, T. M. & Palsson, B. Ø. BiGG: a Biochemical Genetic and Genomic knowledgebase of large scale metabolic reconstructions. *BMC Bioinformatics* **11**, (2010).

12. Ebrahim, A., Lerman, J. A., Palsson, B. O. & Hyduke, D. R. COBRApy: COnstraints-Based Reconstruction and Analysis for Python. *BMC Systems Biology* **7**, (2013).

13. Ross, D. E., Marshall, C. W., Gulliver, D., May, H. D. & Norman, R. S. Defining genomic and predicted metabolic features of the *Acetobacterium* genus. *mSystems* **5**, e00277-20 (2020).

14. Bertsch, J. & Müller, V. CO metabolism in the acetogen *Acetobacterium woodii*. *Appl Environ Microbiol* **81**, 5949–5956 (2015).

15. Koch, S. *et al.* RedCom: A strategy for reduced metabolic modeling of complex microbial communities and its application for analyzing experimental datasets from anaerobic digestion. *PLOS Computational Biology* **15**, e1006759 (2019).

16. Arkin, A. P. *et al.* KBase: The United States Department of Energy Systems Biology Knowledgebase. *Nat Biotechnol* **36**, 566–569 (2018).

17. Lieven, C. *et al.* MEMOTE for standardized genome-scale metabolic model testing. *Nat Biotechnol* **38**, 272–276 (2020).

18. Hucka, M. *et al.* The Systems Biology Markup Language (SBML): Language Specification for Level 3 Version 1 Core. *Journal of Integrative Bioinformatics* **12**, 382–549 (2015).

19. Khandelwal, R. A., Olivier, B. G., Röling, W. F. M., Teusink, B. & Bruggeman, F. J. Community flux balance analysis for microbial consortia at balanced growth. *PLOS ONE* **8**, e64567 (2013).

20. Benito-Vaquerizo, S. *et al.* Modeling a co-culture of *Clostridium autoethanogenum* and *Clostridium kluyveri* to increase syngas conversion to medium-chain fatty-acids. *Computational and Structural Biotechnology Journal* **18**, 3255–3266 (2020).

21. Benito-Vaquerizo, S. *et al.* Model-driven approach for the production of butyrate from CO_2/H_2 by a novel co-culture of *C. autoethanogenum* and *C. beijerinckii*. *Front. Microbiol.* **13**, 1064013 (2022).
22. Benito-Vaquerizo, S. *et al.* Genome-scale metabolic modelling enables deciphering ethanol metabolism via the acrylate pathway in the propionate-producer *Anaerotignum neopropionicum*. *Microb Cell Fact* **21**, 116 (2022).
23. Zou, W. *et al.* Genome-scale metabolic reconstruction and analysis for *Clostridium kluyveri*. *Genome* **61**, 605–613 (2018).
24. Chan, S. H. J., Simons, M. N. & Maranas, C. D. SteadyCom: Predicting microbial abundances while ensuring community stability. *PLOS Computational Biology* **13**, e1005539 (2017).
